# Tangled Evolutionary History: Genetically Divergent Taxa and Hybrids Characterise Lantana Invasions in Australia

**DOI:** 10.1111/eva.70251

**Published:** 2026-05-20

**Authors:** Patricia Lu‐Irving, Francisco Encinas‐Viso, Eilish McMaster, Jason Callander, Michael D. Day, Johannes J. Le Roux

**Affiliations:** ^1^ Research Centre for Ecosystem Resilience Botanic Gardens of Sydney Sydney New South Wales Australia; ^2^ National Herbarium of New South Wales Botanic Gardens of Sydney Mount Annan New South Wales Australia; ^3^ Centre of National Australian Biodiversity Research CSIRO Canberra Australian Capital Territory Australia; ^4^ School of Life, Earth, and Environmental Sciences University of Sydney Sydney Australia; ^5^ Department of Primary Industries Ecosciences Precinct Brisbane Queensland Australia; ^6^ School of Natural Sciences Macquarie University Sydney New South Wales Australia; ^7^ Centre for Invasion Biology Department of Botany and Zoology Stellenbosch University Stellenbosch South Africa

**Keywords:** Australia, biological control, invasive species, *Lantana camara*, population genomics, taxonomy, weeds

## Abstract

This study investigated population genomic diversity and structure in the taxonomically complicated and highly invasive *Lantana camara* L. (Verbenaceae) species complex in Australia. We considered taxonomy, spatial distribution and patterns of morphological and genetic variation in populations across the main invaded area (i.e., coastal and subcoastal eastern Australia across 22° latitude), and reconstructed their relationships with native‐range plants sampled from the Americas. We used DArTseq to generate genome‐wide single nucleotide polymorphism (SNP) data for > 600 individuals and analysed data from > 20,000 SNPs to identify genetic structure, and test the extent to which it corresponded with taxonomic descriptions and morphotype concepts used in current lantana biological control programmes. A subset of individuals was also evaluated for ploidy variation using flow cytometry. We used MaxEnt to predict suitable habitat for invasive lantana in Australia, and compared predictions with current distribution patterns, together with variation in morphology and biological control agent susceptibility. Invasive lantana in Australia was not clearly related to any native‐range *Lantana* species. Instead, it was characterised as a mosaic of several distinct and divergent genetic lineages, from common and widespread to rare and localised, interspersed with occasional inter‐lineage hybrids. This complexity explains why the taxonomy of invasive lantana has been (and will continue to be) challenging. Nevertheless, the main genetic lineages identified here were in agreement with published taxonomic concepts (varieties). Two of these varieties (Common Pink and Common Pink‐Edged Red) were found to be dominant, widespread and occupying the full extent of their predicted suitable habitat in Australia. Failing to distinguish different taxa within invasive lantana may affect the success of biological control; taxonomic revision is thus needed to comprehensively define invasive taxa and provide broadly‐applicable tools to identify them accurately.

## 1 Introduction

Invasive species are a major threat to biodiversity conservation (Kearney et al. 2019; Roy et al. 2024; Vilà et al. 2011). Accurate identification of invasive species is needed to effectively mitigate their impacts, for example, for selection of biological control agents (Castillo et al. 2021; Pyšek et al. 2013), a highly cost‐effective management approach (McFadyen 1998; Schwarzländer et al. 2018; van Wilgen et al. 2012). Some factors that obscure the taxonomy of invasive species can also play a role in their ecological success, for example, multiple species introductions and hybridisation enabling rapid adaptation to new environments (Ellstrand and Schierenbeck 2000; Le Roux and Wieczorek 2009; Schierenbeck and Ellstrand 2009). Therefore, understanding the taxonomy of invasive species is integral in understanding the processes contributing to invasion, and optimising management outcomes (Le Roux 2021; Pyšek et al. 2013). A classic example of problematic taxonomic uncertainty in an invasive species is *Lantana camara* L. (Verbenaceae; Day, Wiley, et al. 2003; Urban et al. 2011).

The *Lantana camara* species complex (*L. camara sensu lato*, hereafter referred to as “lantana”) has been listed among the world's worst invasive species (Lowe et al. 2000); invasive lantana populations have been reported in over 90 countries. Among the range of economic, environmental and human health impacts that lantana has been documented to cause (e.g., AEC Group 2007; Kearney et al. 2019; Silver and Carnegie 2017; Syed and Guerin 2004), it is known to poison livestock. However, taxonomic uncertainty makes toxicity difficult to predict (Everist 1981). In Australia, lantana is listed as one of 32 Weeds of National Significance (Thorp and Lynch 2000) and has invaded millions of hectares over thousands of kilometres of eastern Australia, spanning 22° in latitude. Lantana has the greatest impact of any invasive plant on Australia's threatened species (Kearney et al. 2019) and has been estimated to cause over AU$100 million in economic losses to agriculture annually (AEC Group 2007).

Biological control of lantana has been implemented in many countries, including Australia. However, despite 44 biological control agents released globally since 1902 (30 of these in Australia), lantana is still not considered to be under adequate control (Winston et al. 2025). There are several constraints that may explain the modest success of lantana biological control to date. First, matching invasive plant genotypes with their co‐evolved specialist natural enemies can improve biological control effectiveness (Goolsby et al. 2006), but this strategy depends on knowing the native origins of invasive populations (Jourdan et al. 2019), and these are poorly understood for invasive lantana populations worldwide. Second, closely tied to native provenance, is accurate taxonomic identification of invasive populations and their associated bioclimatic niches. In addition to historical adaptation of natural enemies to their host plants, successful biological control depends on the invaded environment being suitable for agent establishment and spread (McFadyen 1991). Thus, unresolved taxonomy and biogeography are among the main factors limiting successful biological control of lantana (Day and Neser 2000).

Invasive and cultivated forms of lantana are derived from a clade of up to 20 species naturally found from southern North America to southern South America (*Lantana* section *Lantana*; Lu‐Irving et al. 2021; O'Leary et al. 2023; Sanders 2006; Sanders 2012). While the name *Lantana camara* is routinely applied to invasive and cultivated members of section *Lantana*, their wide range of morphological variation does not align with Linnaeus' (1753) concept of *L. camara* , nor with any other naturally occurring *Lantana* species (Sanders 2006, 2012; Smith and Smith 1982; Urban et al. 2011). This is consistent with an origin in, and history of, cultivation: different species of *Lantana* were extensively hybridised to create ornamental cultivars, particularly in the mid to late 19th century (Howard 1969), when introductions to Australian gardens were likely to have taken place. Ornamental lantana was cultivated in multiple areas in Australia by 1850, and its invasiveness was noted by the end of the 19th century (Michael 1987; Swarbrick 1986).

Invasive lantana comprises extensive morphological diversity and variation in ploidy (Day, Wiley, et al. 2003; Spies 1984; Urban et al. 2011), consistent with inter‐specific hybridisation having played a major role in its history. Previous attempts to make sense of this variation include multiple, often competing, classification systems that range from formal names and descriptions (Munir 1996; Sanders 2006, 2012; Smith and Smith 1982) to less formal working concepts (Day, Broughton, and Hannan‐Jones 2003; Heystek 2006). The lack of a well‐supported taxonomic treatment for invasive lantana has resulted in weed scientists, managers, and legislators using a broad species concept (i.e., applying the name *Lantana camara* to the entire complex without recognising infraspecific taxa) despite generally acknowledging the taxonomic uncertainty involved in this approach (e.g., Johnson 2007; Thorp and Lynch 2000). Treating lantana as a single, albeit highly variable species invites certain assumptions that are untested and may be invalid (e.g., all individuals are fully interfertile, morphological variants are not genetically divergent). Whether lantana is treated as a single taxon or as multiple taxa has important management implications: while broad assessments and chemical or mechanical control can proceed under a single‐taxon framework, effective biological control and locally‐specific assessment of impact (e.g., toxicity to livestock) and weed risk (e.g., potential to spread) require a clear understanding of the taxonomic makeup of invasive populations (Marvaldi 2024; Rosen 1986).

Australian biological control researchers have used the informal concept of five ‘varietal groups’ to identify lantana variants that represent putatively distinct target hosts (Day, Broughton, and Hannan‐Jones 2003). These groups (hereafter referred to as morphotypes) were based on aggregating the 29 varieties described by Smith and Smith (1982) according to flower colour: pink, red, pink‐edged red, orange and white. However, multiple biological control agents have failed to establish on some morphotypes, and establishment has been inconsistent with morphotype identity in Australia (Day, Wiley, et al. 2003; Day, Broughton, and Hannan‐Jones 2003). This may be attributable to mismatches between agents and target hosts, and/or unsuitable bioclimatic conditions for agent establishment in invaded ranges (Day and Neser 2000; McFadyen 1991). That is, the five‐morphotype concept may fail to capture underlying variation in Australian lantana, and/or some morphotypes may be invading areas that are bioclimatically unsuitable for their natural enemies. There is a clear need to identify biologically meaningful variation within invasive lantana in Australia, and this need is still outstanding despite multiple attempts to address it. Traditional DNA sequencing analyses have found insufficient variation to resolve evolutionary relationships within *Lantana* sect. *Lantana*; while dominant genetic markers (e.g., RAPD, AFLP) showed more success, studies using these approaches lacked sufficient data and/or taxon sampling to yield informative insights into the invasive complex (Lu‐Irving et al. 2021; Scott et al. 1997; Watts 2009). Recently, population‐level genotyping‐by‐sequencing approaches (with much greater genomic coverage and denser geographic sampling) have shown promise (Lu‐Irving et al. 2022; Praveen et al. 2025).

**FIGURE 1:**
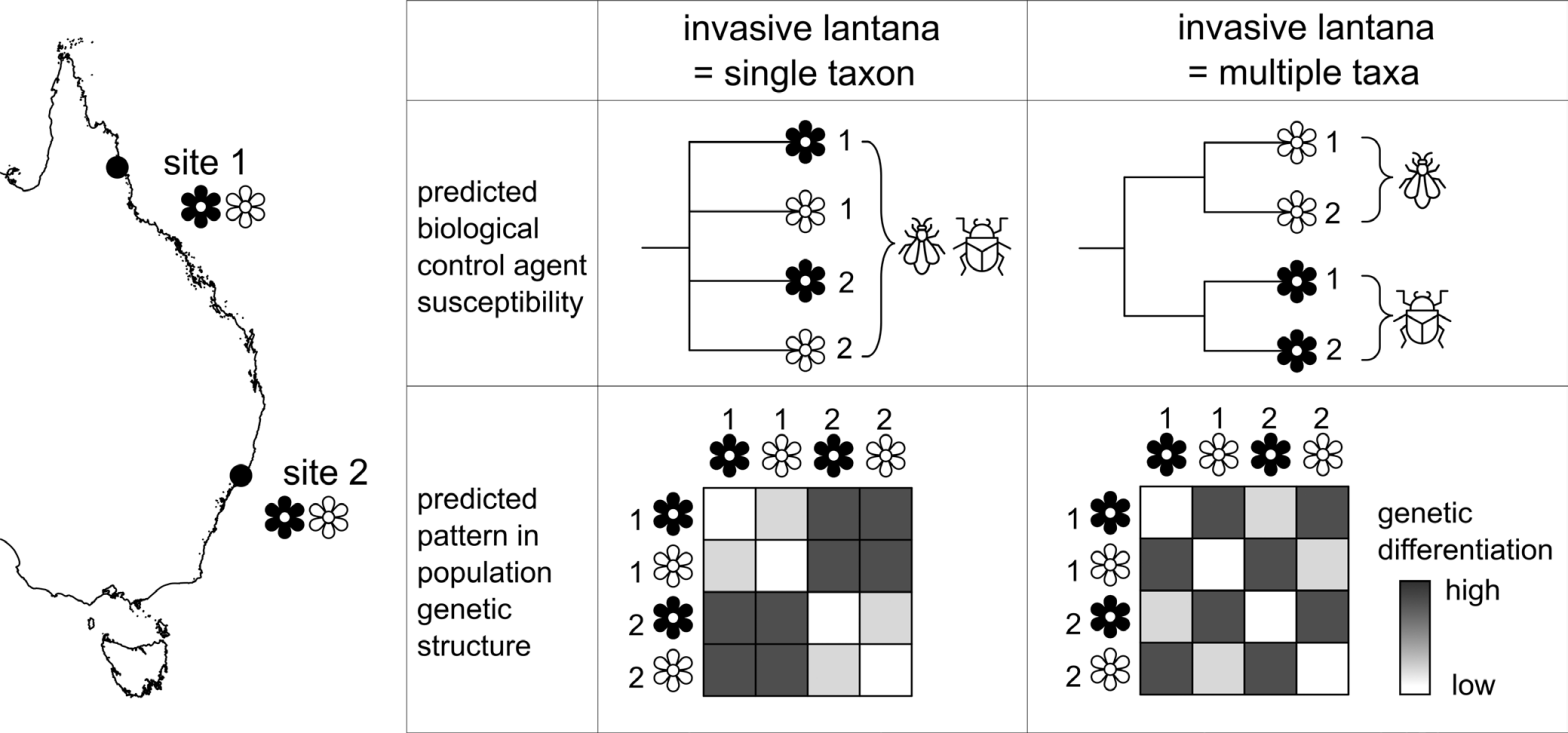
Conceptual diagram summarising taxonomic hypotheses and predictions for invasive lantana in Australia. This example shows two different lantana flower colour morphotypes (denoted by black and white) co‐occurring at each of two geographically distant sites. If invasive lantana comprises only a single natural taxon (i.e., plants with different flower colours do not belong to genetically differentiated lineages; null hypothesis), it is predicted that biological control agents will show no preferences for different host morphotypes, and co‐occurring plants will share more alleles with each other than with geographically distant plants regardless of morphotype. However, if invasive lantana consists of distinct and divergent genetic lineages corresponding with flower colour (warranting recognition as multiple taxa; alternate hypothesis), it is predicted that different biological control agents will prefer different host morphotypes, and that plants of the same morphotype will share more alleles with each other than with plants of different morphotypes regardless of geographic distance.

**FIGURE 2:**
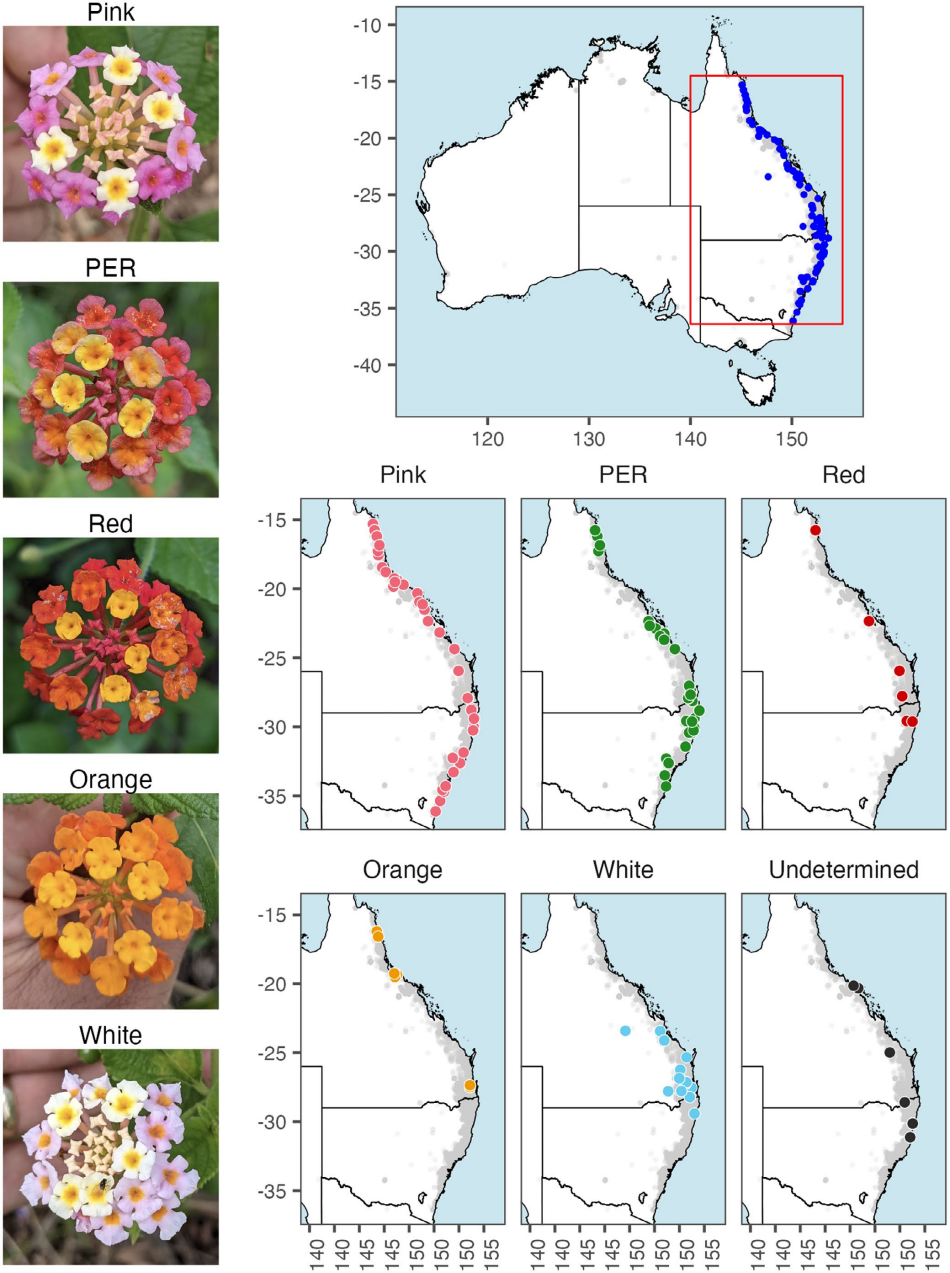
Flower morphotypes and distribution of lantana in Australia. Photographs of each of five flower colour morphotypes (varietal groups) recognised by lantana biological control researchers in Australia (Day, Broughton, and Hannan‐Jones 2003), and maps showing their sampled distributions along the continent's eastern coast. Photographs (top to bottom): Inflorescences of individuals identified in the field as pink, pink‐edged red (PER), red, orange, white flower colour morphotypes. All maps show pale grey points representing the current distribution of lantana in Australia: Vouchered occurrence records for *Lantana camara* collected between 2000 and 2025, obtained from the Australasian Virtual Herbarium. Additionally plotted are points showing all locations sampled in this study (dark blue; top), and locations where samples were identified to morphotype or otherwise unable to be identified (six individually coloured maps; bottom).

In this study, we collected samples and molecular data on an unprecedented scale to deliver new insights into the genetic variation and structure as well as the evolutionary history of lantana in Australia. We used DArTseq to discover thousands of single nucleotide polymorphisms (SNPs) across hundreds of lantana individuals from the native range in the Americas and the invaded range in Australia. Since variation in polyploidy might provide insights into patterns of hybridisation and gene flow, we tested for ploidy variation by estimating the genome sizes of a subset of individuals using flow cytometry. Our overall aim was to characterise patterns in genetic diversity to test whether *L. camara s.l*. in Australia represents a homogeneous genetic lineage (i.e., best treated as a single taxon), versus multiple, independent lineages (taxa) maintained by some degree of reproductive isolation (Figure 1). More specifically, we tested the extent to which genetic structure within lantana, if present, would align with the five flower colour morphotypes proposed by Day, Broughton, and Hannan‐Jones (2003); (Figure 2), and reconstructed phylogeographic relationships between Australian invasive lantana and native‐range *Lantana* sect. *Lantana*. Finally, we used our genotyped sample occurrence data to explore how the concept of a single taxon versus multiple taxa might affect species distribution modelling (SDM) to predict biological control agent success and future weed risk.

## 2 Methods

### 2.1 Sampling

Wild plants belonging to *Lantana* section *Lantana* were sampled in the field: leaf tissue was collected and preserved in silica gel and/or by lyophilisation. In the invaded range in eastern Australia, a sample from each of six individuals (> 10 m apart where possible) was collected (with permission) at each site to maximise the capture of genetic diversity within and among sites. Where multiple morphotypes co‐occurred at a site, six individuals per morphotype were sampled to test the hypothesis that they would represent separate lineages (i.e., multiple taxa; Figure 1). Additional representation of both native and Australian ranges was achieved by sampling from specimens at Nagoya‐compliant institutions: the Australian National Herbarium (CANB) and the Burke Museum Herbarium (WTU). In total, 537 individuals from 90 sites representing the invasion in eastern Australia and 166 individuals from the native range of *Lantana* section *Lantana* (Argentina, Belize, Bolivia, Brazil, Colombia, Costa Rica, Dominican Republic, Ecuador, Guatemala, Jamaica, Mexico, Peru, USA, Venezuela) were sequenced (Figure 2, Table S1).

All five morphotypes based on flower colour as described by Day, Broughton, and Hannan‐Jones (2003) were collected from multiple sites, with sampling proportions per morphotype of 36% pink, 34% pink‐edged red, 11% white, 7% orange, 6% red and 5% undetermined. Where unusual morphotypes were encountered in the field, we sampled these. We tried to sample as evenly as possible among the five morphotypes, but we observed that their relative abundance in the wild was highly uneven, as previously reported by Day, Broughton, and Hannan‐Jones (2003), that is, pink was the most common flower colour, followed by pink‐edged red, with other morphotypes relatively uncommon at the national scale.

### 2.2 Sample Identification

Smith and Smith (1982) described 29 varieties of *Lantana camara* in Australia, with multiple varieties of each flower colour. Distinctions between them were often subtle and/or subjective (e.g., relying on different shades or hues of colour) and sometimes based on overlapping ranges of measurements rather than categorical traits, making it challenging to identify plants to one of the 29 varieties. Due to the difficulty using Smith and Smith's (1982) treatment for identification, especially with pressed specimens (since the colour and shape of the corolla are both lost after pressing), we did not attempt to identify samples to varietal level. Instead, we made post hoc comparisons between Smith and Smith's (1982) descriptions and field‐collected specimens identified as belonging to different genetic lineages (see Results).

Sanders (2012) produced a taxonomic treatment of *Lantana* section *Lantana* focused mainly on the native range in which he recognised 20 species (including 13 subspecies and five varieties), distinguished mainly by qualitative differences in leaf hair morphology. Identification of specimens this way was challenging due to the subtlety and subjectivity in interpreting the variation in this character, but some herbarium specimens were identified to species described by Sanders (2012) with the assistance of Sanders himself. Some specimens were morphologically determined to be non‐hybrid individuals of the following nine species of *Lantana* section *Lantana* (the clade from which the invasive *L. camara* *s.l*. complex is derived): *L. bahamensis* Britton, *L. camara* L., *L. depressa* Small, *L. horrida* Kunth, *L. leonardiorum* Moldenke, *L. nivea* Vent., *L. scabrida* Aiton, *L. strigocamara* R.W. Sanders, *L. urticoides* Hayek. However, most specimens, particularly Australian specimens, were determined to be putative hybrids (Table S1).

### 2.3 Genotyping

We used the marker‐discovery pipeline developed by Diversity Arrays Technology Pty Ltd. (DArT), which is comparable to ddRADseq (Peterson et al. 2012), and has been successfully applied in various systems including non‐model and polyploid plants (Kilian et al. 2012).

Approximately 5 mg of desiccated leaf tissue per sample was sent to DArT (Canberra, Australia) for DNA extraction and DArTseq analysis using previously described approaches (Jaccoud et al. 2001; Kilian et al. 2012; Mao et al. 2020; Sansaloni et al. 2011; Wenzl et al. 2004). Briefly, genomic DNA was extracted using a plant‐specific modified CTAB protocol (Doyle and Doyle 1987) and checked for quality prior to complexity reduction by digestion with restriction enzymes *Pst*I and *Ase*I, fragment size selection and Illumina short‐read sequencing. Reads were processed and SNPs were called using DArT proprietary analytical pipelines, including the use of technical replicates to score the reproducibility of each locus. Data were provided as biallelic codominant SNP genotype matrices, and metadata describing marker quality.

Of the 703 samples of lantana submitted for SNP discovery, genotype data were obtained for 687 samples; 16 samples did not yield usable data (Table S1). Following processing and filtering of SNP matrices, two datasets were generated for further analysis: (1) 512 eastern Australian samples by 10,078 loci for population genetic analyses (4986 loci after filtering with minor allele frequency (MAF) ≥ 2%); (2) 524 eastern Australian samples and 128 native range samples (652 total samples) by 25,751 loci for phylogenetic inference (6462 loci after filtering with MAF ≥ 2%). The two different datasets were assembled and analysed differently according to the different aims of each: the first used analyses to maximise insight into the population‐level patterns of genetic diversity in the lantana invasion of eastern Australia, and the second was focused on reconstructing phylogeographic relationships between Australian and native‐range genetic lineages.

### 2.4 Genome Size Estimation

Flow cytometry was used to measure and compare genome size as an indicator for ploidy variation among 14 individuals of the pink and pink‐edged red morphotypes from five sites. *Glycine max* was used as an internal standard. Whole nuclei were extracted from fresh leaf tissue following standard protocols. Briefly, up to 1 cm^2^ of leaf tissue was chopped in Sysmex Cystain PI Abs. P extraction buffer and filtered before staining with Sysmex Cystain PI Abs. P staining buffer and incubating at room temperature for 30–60 min. Samples were analysed using a BD Accuri flow cytometer.

### 2.5 Population Genetic Diversity and Structure of Australian Lantana

To better understand patterns of genetic diversity and relationships among invasive lantana populations in eastern Australia, we analysed a subset of the data consisting of only samples collected in the states of Queensland (Qld) and New South Wales (NSW). All analyses were conducted in the R statistical environment (R v4.1.0; R Core Team 2021). To ensure data quality, loci with reproducibility < 98% or missing data > 80% were removed. Fixed loci were excluded, and only one SNP per DArT tag was retained. Samples with > 80% missing data were discarded. The permissive missing‐data threshold was used to retain loci that, while not shared across all divergent genetic lineages, provide valuable information on within‐group variation and population structure and improve the resolution of evolutionary relationships (e.g., Tripp et al. 2017). Downstream analyses (e.g., PCA, diversity metrics and pairwise *F*_ST_) further subset loci to those informative for the specific comparisons, using a more stringent filter for missing data. Prior to conducting Principal Component Analysis and calculating diversity statistics, loci were further filtered to retain only those with a MAF of ≥ 2%. After filtering, this dataset consisted of 512 samples by 10,078 loci (4986 loci after filtering with MAF ≥ 2%).

Principal Component Analysis (PCA) was conducted using the *adegenet* package (v2.1.10; Jombart 2008). To identify genetic clusters, t‐distributed stochastic neighbour embedding (t‐SNE) was performed. The genotype data was converted to a Euclidean distance matrix, and the t‐SNE algorithm was run using the *Rtsne* R package (v0.17) with parameters set to a perplexity of 15, and a theta of 0. Clusters were subsequently identified by applying hierarchical density‐based spatial clustering (HDBSCAN) using the *dbscan* v1.1–12 R package (Hahsler et al. 2019; Scitovski and Sabo 2020) with a minimum points parameter of 5. Clusters containing fewer than 10 samples were removed, as were clusters that were not monophyletic (as assessed using the Unweighted Pair Group Method with Arithmetic mean (UPGMA) clustering on Euclidean distances).

To evaluate patterns of genetic structure, ancestry estimation was conducted using sparse non‐negative matrix factorization (sNMF) implemented in the *LEA* R package (v3.14.0; Frichot and François 2015) across a range of K values (1–20) with default settings.

Pairwise fixation indices (*F*_ST_) between populations and genetic lineages were calculated using the relative beta estimator (Weir and Hill 2002) with the *SNPrelate* package (v1.20.1; Zheng et al. 2012). Analyses included only shared loci filtered to allow a maximum of 80% missing data and a MAF greater than 2%. Genetic lineages were identified as monophyletic HDBSCAN clusters (see Results). A linear model was used to test if *F*_ST_ differences between populations were influenced by genetic lineage membership and pairwise geographic distance. The analysis was performed using the *stats* R package (v4.3.1).

Genetic diversity was assessed using basic diversity statistics: observed heterozygosity (*H*_*O*_) and inbreeding coefficient (*F*_*IS*_) (Keenan et al. 2013).

#### 2.5.1 Identification of Putative Hybrids

Most samples could be identified in the field according to the five flower colour morphotypes. Notable exceptions were plants at two NSW sites (Korora and Nana Glen), with flowers that were intermediate between pink and pink‐edged red. The intermediate morphotype at both sites was sympatric with both pure morphotypes (i.e., pink‐ and pink‐edged red‐flowered plants), and intermediate plants were hypothesised to be hybrids. To test this hypothesis, we investigated pure and intermediate morphotypes at these two sites and at two nearby sites where only pure morphotypes occurred by (1) comparing observed heterozygosity with the results of PCA for these individuals, (2) visualising patterns in differentially fixed vs. shared alleles, (3) estimating posterior probabilities of putative hybrids and their parental classes using NewHybrids (Anderson and Thompson 2002). For the NewHybrids analysis, we subset the Australian‐only dataset to include only the target individuals, then filtered loci to a maximum missingness of 20%, removed fixed SNPs, and applied a MAF filter of 1%. From the remaining loci, 400 were randomly selected for analysis. We then ran the gl.nhybrids function from the dartR v 4.3.1 R package (Mijangos et al. 2022) in unsupervised mode (no parental groups specified), with a burn‐in of 100 iterations and 100 sweeps.

### 2.6 Relationships Among Australian and Native‐Range Lantana

To infer evolutionary relationships among samples from eastern Australia and the native range, we used two distance‐based approaches. Loci were filtered as described above.

A phylogenetic network was constructed in SplitsTree v4.18.2 (Huson and Bryant 2006) to estimate relationships among lineages while allowing for reticulation (e.g., historical hybridisation), using a Euclidean distance matrix and the *RSplitsTree* v0.1.0 package. The network was plotted in R using *tanggle* v1.8.0 package (Schliep et al. 2024).

A dichotomously‐branching phylogenetic tree was estimated using the Unweighted Pair Group Method with Arithmetic mean (UPGMA), which bypasses assumptions of model‐based approaches, which are problematic in this case due to sparse SNPs, lack of reference genomes and the potential effects of hybridisation. A Hamming distance matrix was used to generate UPGMA dendrograms, with bootstrap support (10,000 replicates) calculated using the *phangorn* v2.11.1 package (K. P. Schliep 2011) to assess the topology's robustness.

### 2.7 Species Distribution Modelling

To investigate how different environmental variables might influence the distribution of different genetic lineages of invasive lantana in Australia (i.e., the concept of multiple distinct taxa), we analysed sample occurrence data to predict habitat suitability of each genetic lineage (see Results) separately, as well as of all samples combined.

Habitat suitability was estimated with species distribution models using the maximum‐entropy (MaxEnt) method based on the environmental variables of sample collection coordinates. Species distribution models were constructed using the MaxEnt v3.4.1 software (Phillips et al. 2017) and the *dismo* v1.3–3 R package (Hijmans et al. 2024) using all specimen records as training data, 21 environmental variables as predictor layers at 1 km^2^ resolution, and 10,000 background points. Specimen records corresponded with field collection coordinates for samples falling into identified genetic lineages (see Results; Figure S2).

The environmental predictors used for the MaxEnt models were 15 bioclimatic variables and six soil and landform variables. The bioclimatic variables were: temperature seasonality (CV; B04), maximum temperature of warmest month (°C; B05), minimum temperature of coldest month (°C; B06), mean temperature of warmest quarter (°C; B10), mean temperature of coldest quarter (°C; B11), annual precipitation (mm; B12), precipitation seasonality (CV; B15), precipitation of driest quarter (mm; B17), annual mean moisture index (B28), moisture index seasonality (CV; B31), mean moisture index of wettest quarter (Bio32), annual total actual evapotranspiration (mm; EAA), minimum monthly potential evaporation (mm; EPI), minimum monthly atmospheric water deficit (mm; WDI) and maximum monthly atmospheric water deficit (mm; WDX). Soil and landform variables were: soil clay content 0–30 cm (%; CLY), soil sand content 0–30 cm (%; SND), total soil nitrogen 0–30 cm (%; NTO), total soil phosphorus 0–30 cm (%; PTO), soil bulk density 0–30 cm (g/cm^3^; BDW) and topographic wetness index (TWI3S). Data (9 s resolution) are available from the CSIRO Data Access Portal at https://doi.org/10.25919/5dce30cad79a8 (Bio4‐Bio32), https://doi.org/10.4225/08/5afa9f7d1a552 (EAA, EPI, WDI and WDX) and https://doi.org/10.4225/08/5b285fd14991f (CLY, SND, NTO, PTO, BDW and TWI3S). To remove highly correlated environmental variables (> 0.7), we quantified the correlation matrix using the function “findCorrelation” from the *caret* R package (Kuhn 2008). Following this, we retained nine environmental predictors (B04, B31, BDW, CLY, EAA, EPI, PTO, TWI3S, WDI) for further analyses. The Cloglog transform as a continuous index of habitat suitability was selected as the output format (Phillips et al. 2017). The maximum of Cohen's Kappa (K, which determines the optimal threshold for statistical discrimination of presence‐absence; Cohen 1960), the maximum training sensitivity plus specificity (MTSS, equivalent to finding the point in the receiver operator characteristic (ROC) curve with a tangent slope of 1; Cantor et al. 1999) and the area under the ROC curve (AUC) were applied to test model performance.

## 3 Results

### 3.1 Genome Size Estimation

Genome size estimates obtained via flow cytometry ranged from 6.08 to 6.89 pg (2C), with 2C DNA content of pink‐flowered plants on average 0.27 pg greater than pink‐edged red plants (Figure S1). These estimates are broadly consistent with previously published genome size estimates in tetraploid *Lantana* Sect. *Lantana* (6.08–6.42 pg; Brooks Parrish et al. 2021; Deng et al. 2017).

### 3.2 Population Genetic Diversity and Structure of Australian Lantana

**FIGURE 3:**
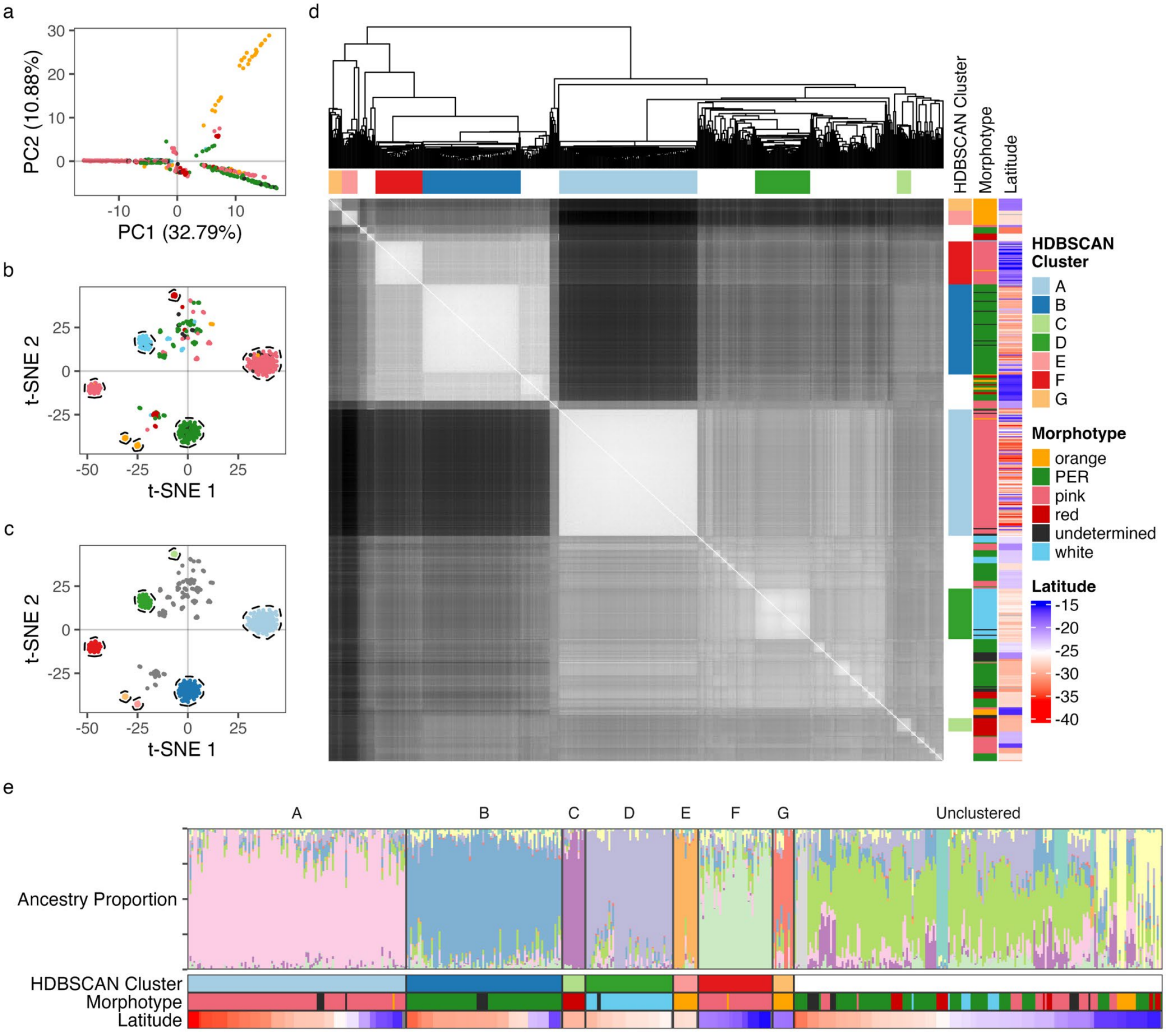
Population genetic diversity and structure of Australian lantana. Analyses of genetic similarity, clustering and structure among 512 wild *Lantana camara s.l*. samples from eastern Australia. (a) plot of first two Principal Components identified by PCA (points coloured by morphotype); (b) t‐SNE plot (points coloured by morphotype) with clusters identified by HDBSCAN circled; (c) t‐SNE plot as in (b), but here points coloured by HDBSCAN cluster; (d) Plot showing a heatmap of pairwise genetic (Euclidean) distances between samples, ordered by UPGMA clustering (dendrograms shown). Blocks of genetically similar samples (lighter shades) align well with HDBSCAN clusters and with morphotypes, but only partially with collection latitude (indicated by adjacent annotations). (e) Plot showing proportion/ancestry coefficient of each sample (vertical bars) for each of *K* = 11 inferred ancestral populations (LEA snmf). Samples are grouped by HDBSCAN clusters, with morphotype and latitude annotations below; patterns in ancestry coefficient align with major genetic lineages and with morphotype but only partially with latitude.

Principal Component Analysis (PCA) revealed patterns among Australian lantana samples that partially aligned with morphotypes (Figure 3a), but did not clearly separate them. Dimensionality reduction using t‐SNE produced more distinct subgrouping (Figure 3b–c). Clustering on t‐SNE with HDBSCAN identified seven clusters, equivalent to genetic lineages (groups with ≥ 10 individuals; monophyletic in UPGMA), labelled A–G (Figure 3c–e). Of 512 Australian samples analysed, 62% were assigned to one of the seven genetic lineages (Table 1). Flower colour morphotypes were consistent within lineages but not diagnostic of lineages, because two morphotypes each encompassed two lineages: plants with pink flowers (36% of samples) comprised lineages A and F, while orange‐flowered plants comprised lineages E and G. The t‐SNE with HDBSCAN method groups samples by genetic similarity, requiring a minimum density of similar individuals to form a cluster. Samples that were genetically intermediate, highly variant, or located at cluster edges lacked sufficient genetic similarity with other samples to be grouped using this method. These unclustered samples (38% of samples) included all morphotypes and were distributed throughout the geographic area we sampled.

**TABLE 1:** Morphotype distribution across genetic lineages.

		Morphotype						Total	Variety	Origin of closest native‐range relative
		Pink	PER	Red	Orange	White	Undetermined			
Lineage	A	109	0	0	1	0	5	115 [22%]	Common Pink	Brazil
	B	0	76	0	0	0	6	82 [16%]	Common Pink‐Edged Red	Mexico
	C	0	0	12	0	0	0	12 [2%]	Oblong Red	—
	D	0	0	0	0	44	2	46 [9%]	Helidon White	—
	E	0	0	0	13	0	0	13 [3%]	True Orange	—
	F	38	0	0	1	0	0	39 [8%]	Townsville Red‐centred Pink	Mexico
	G	0	0	0	11	0	0	11 [2%]	Townsville Prickly Orange	—
	Unclustered	39	96	21	10	13	15	194 [38%]		
Total		186 [36%]	172 [34%]	33 [6%]	36 [7%]	57 [11%]	28 [5%]	512		

*Note:* Morphotype distribution across genetic lineages in invasive lantana in eastern Australia. The table shows the number of samples for each morphotype (pink, pink‐edged red (PER), red, orange, white, undetermined) within seven genetic lineages (A–G) and samples that were unclustered. Each lineage is associated with a flower colour morphotype, a varietal name described by Smith and Smith (1982), and the origin of the closest native‐range relative where known. Percentages reflect the proportion of samples in each lineage relative to the total dataset (*n* = 512).

**FIGURE 4:**
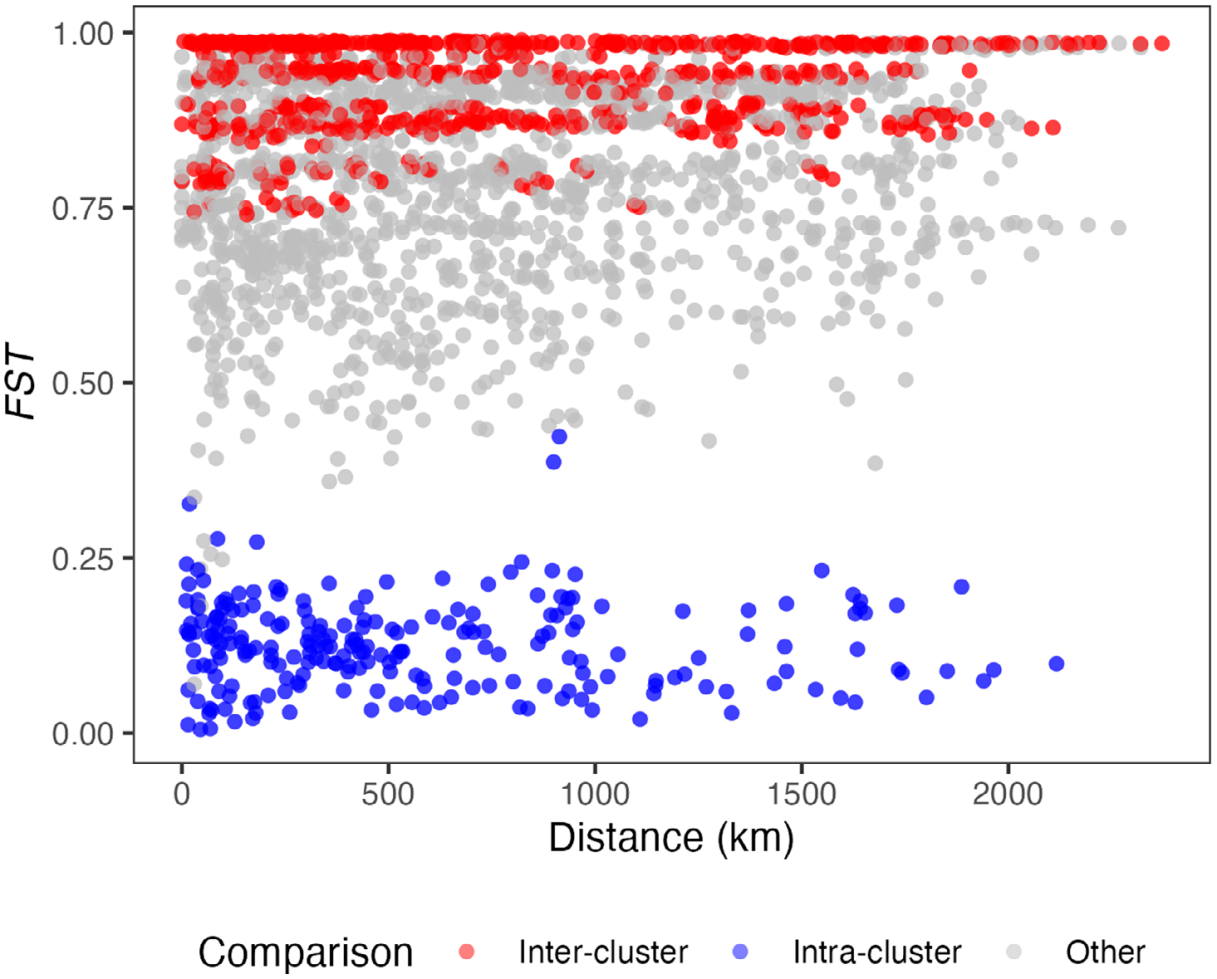
Pairwise *F*_ST_ values and geographic distance between lantana collection sites in eastern Australia. Pairwise comparisons within the same genetic lineage are coloured blue (intra‐cluster), comparisons between different genetic lineages are red (inter‐cluster), and comparisons involving unclustered sites are grey (other). All sites consist exclusively of samples from a single genetic lineage or unclustered samples. Linear model results showed that both genetic lineage membership (inter‐cluster, intra‐cluster, other) and geographic distance had statistically significant effects on pairwise *F*_ST_ (*p* < 2e‐16 for all variables).

The seven genetic lineages identified by t‐SNE and HDBSCAN ranged from common and widespread to infrequent and geographically restricted. They were strongly genetically differentiated regardless of geographic proximity (Figure 4) and varied in within‐lineage genetic diversity. Each lineage corresponded in morphotype and distribution to a variety described by Smith and Smith (1982). Lineage information is summarised in Table 1; see also Figure 3d (within‐ vs. between‐lineage genetic similarity), Figure S2 (lineage distributions), Figure S3 and Tables S3 and S4 (heterozygosity). Lineage A (*n* = 115 pink‐flowered individuals) was widespread from northern Qld to southern NSW, and matched the description and distribution of the Common Pink variety described by Smith and Smith (1982). Lineage A individuals had low observed heterozygosity (0.015 ± 0.004) and were highly genetically similar to one another. Lineage B (*n* = 82 pink‐edged red‐flowered individuals) occurred mainly in NSW but also in Qld, and matched the variety Common Pink‐edged Red; these individuals also had low observed heterozygosity (0.003 ± 0.003) and high genetic similarity. Lineage C (*n* = 12 red‐flowered individuals) comprised only two sites in northern NSW, matched Oblong Red and had high observed heterozygosity (0.153 ± 0.02). Lineage D individuals (*n* = 46, white) matched Helidon White, and also had high observed heterozygosity (0.12 ± 0.022). Smith and Smith (1982) recorded this variety only in southeast Qld, but we found it to be abundant in northern NSW as well. Lineage E individuals (*n* = 13, orange) were only found in southeast Qld, matched True Orange and had high observed heterozygosity (0.151 ± 0.034). Lineage F individuals (*n* = 39, pink), limited to northern Qld, matched Townsville Red‐centred Pink, had low observed heterozygosity (0.019 ± 0.008). Lineage G individuals (*n* = 11, orange), also in northern Qld, matched Townsville Prickly Orange had variable levels of observed heterozygosity (0.06 ± 0.045).

Results of LEA snmf analysis were used to estimate ancestry and further explore genetic structure. These results were not used to define genetic lineages, but were compared with the results of the t‐SNE and HDBSCAN cluster analysis. Ancestry patterns largely aligned with HDBSCAN clusters, and *K*‐values > 7 revealed substructure within the ‘unclustered’ group (Figures 3e and S4); since these additional putative genetic lineages had < 10 samples they were not captured by HDBSCAN. Across tested *K*‐values, *K* > 10 best fit the data (minimised cross‐entropy; Figure S4). Figure 3e shows *K* = 11 to best visualise patterns in genetic structure, but this should not be interpreted as the ‘true’ number of ancestral lineages given the exploratory nature of the analysis (Meirmans 2015) and the possibility of unsampled lineages.

Pairwise *F*_ST_ values between eastern Australian lantana populations (where putative population = morphotype × site; see Methods) revealed strong differentiation between genetic lineages (Figure 4). These results were not used to define lineages but to assess gene flow within and between them. Estimated *F*_ST_ between populations within lineages was consistently low to moderate (< 0.25), indicating limited differentiation regardless of geographic separation. Between lineages, *F*_ST_ was consistently high (> 0.75), indicating limited gene flow even when populations were proximate or sympatric. Estimates involving populations of unclustered samples showed variable *F*_ST_ (but typically > 0.25), suggesting at least some differentiation between populations and from identified genetic lineages. While these estimates may appear high compared with other datasets (*F*_ST_~0.25 has generally been interpreted as strong differentiation, vs. relatively weak in this study), this is typical for analyses of DArT data (McMaster et al. 2024). The linear model showed that both lineage membership and geographic distance significantly influenced pairwise *F*_ST_ comparisons (*p* < 2e‐16 for all variables). Pairwise population *F*_ST_ estimates were lowest within lineages (mean 0.112), highest between lineages (mean 0.904), and high for comparisons involving unclustered samples (mean 0.767). Geographic distance was positively correlated with *F*_ST_, but with a very small effect size (+0.00003 per km). The model explained 78.18% of the variation in pairwise *F*_ST_; F(3, 4966) = 5932, *p* < 2.2e‐16, *R* ^2^ = 0.7818.

#### 3.2.1 Identification of Putative Hybrids

Plants at two sites with flowers that were intermediate in colour between pink and pink‐edged red showed higher individual heterozygosity compared with co‐occurring individuals with pure pink or pink‐edged red flowers (lineages A and B), as would be expected for hybrids compared with their parents; these highly heterozygous individuals were also intermediate between samples of the two putative parental lineages on PC1 (which explained 32.79% of the variation in the PCA; Figure S2). Morphological intermediates were predominantly heterozygous at loci that were reciprocally fixed in lineages A and B (Figure S5b). The unsupervised NewHybrids analysis suggested that morphological intermediates were most likely to be F2 hybrid offspring of the pure morphotypes (Table S5). All these findings were consistent with morphologically intermediate individuals resulting from hybridisation between genetic lineages A and B.

### 3.3 Relationships Among Australian and Native‐Range Lantana

**FIGURE 5:**
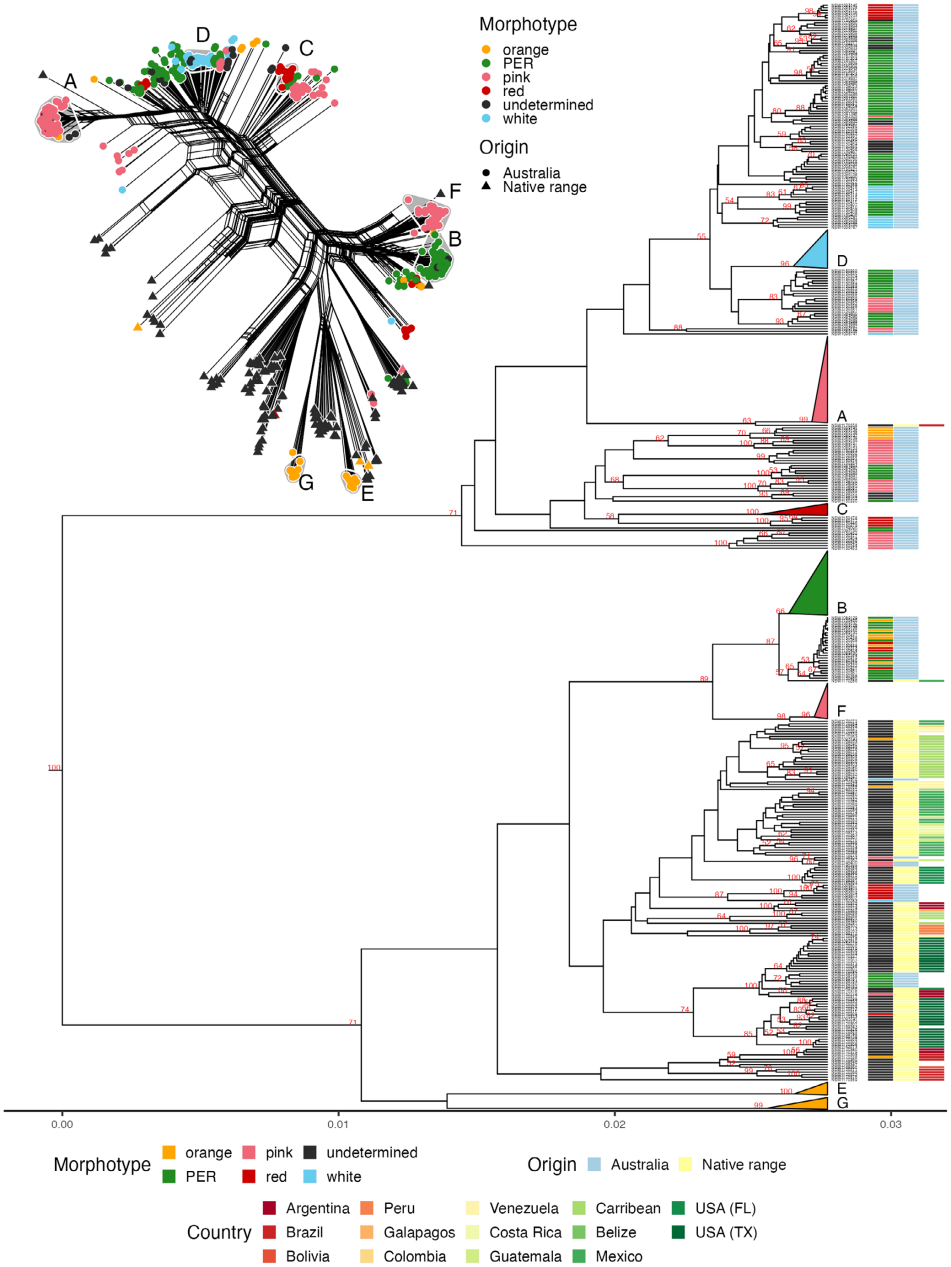
Phylogenetic relationships of Australian and native range lantana. Phylogenetic network (top left) and UPGMA tree depicting the Hamming distance between samples of *L. camara s.l*. from eastern Australia and native‐range members of *Lantana* section *Lantana*. In the network, taxa are coloured according to morphotype and their origins are indicated by shape (circle = Australia, triangle = native range). Samples corresponding with the seven main Australian genetic lineages (= monophyletic genetic clusters as identified by HDBSCAN and UPGMA) are shaded and labelled A–G. The UPGMA tree shows bootstrap values > 50; clades corresponding with Australian genetic lineages are collapsed and labelled A–G. Morphotype and origin are indicated for tips representing unclustered samples.

The seven genetic lineages identified from analysis of the Australian‐only dataset were also recovered in the phylogenetic network and as clades in the UPGMA tree reconstructed from Australian and native‐range samples (Figure 5). The phylogenetic network displayed extensive webbing between groups, suggesting some degree of reticulate evolution (e.g., historic hybridisation). Similarly, the UPGMA topology had poor bootstrap support at deeper nodes, reflecting lack of resolution (to be expected when bifurcating trees are inferred to represent non‐bifurcating, i.e., reticulate, evolutionary relationships).

Most native range samples did not group closely with any Australian samples, and Australian genetic lineages were not closely related to any of the putatively pure native‐range species represented in our analysis. However, lineage A was closely related to a single unidentified sample from southern coastal Brazil, lineage B was related to a single sample from Mexico, and lineage F was related to another sample from Mexico. In contrast, lineages D and C showed no close relationship to any native range samples. Lineages E and G nested closely with native‐range samples in the phylogenetic network, but no sister relationships with specific taxa or samples were recovered in the UPGMA tree.

Several unclustered Australian samples showed closer relationships with native range samples than the majority of Australian samples, for example, six pink‐edged red‐flowered plants from Mount Thorley, NSW, were closely related to plants from Texas, USA; three pink‐flowered plants from near Townsville, Qld were closely related to a Guatemalan plant; one white‐flowered plant from near Emerald, Qld, was closely related to plants from the Dominican Republic. Additionally, six samples from red‐flowered plants near Booubyjan, Qld, and one sample from a white‐flowered plant near Brisbane, Qld, clustered more closely with native‐range samples than other Australian samples.

### 3.4 Species Distribution Modelling

Model performance evaluated using the area under the receiver operating characteristic curve (AUC) was high for all genetic lineages (AUC ≥ 0.97; Table S2). The most important variables for predicting the suitability of habitats for the seven genetic lineages were annual total actual evapotranspiration (EAA), moisture index seasonality (B31), minimum monthly atmospheric water deficit (WDI) and temperature seasonality (°C; B04) (Table S2); response curves to environmental predictors are provided as Supporting Information.

**FIGURE 6:**
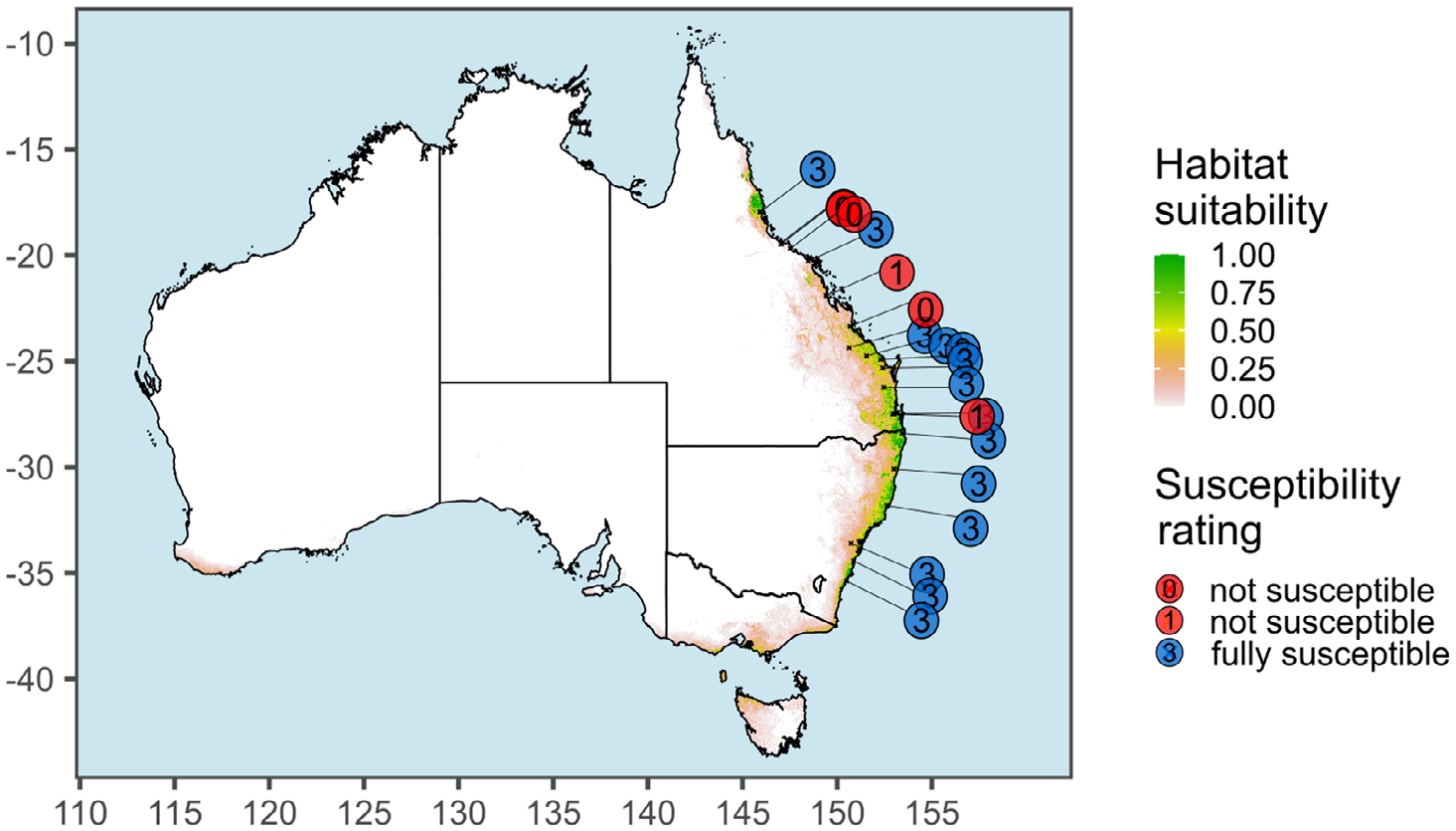
Predicted distribution of Common Pink lantana and susceptibility of pink‐flowered plants to rust. Predicted species distribution under current climatic conditions based on genetically‐verified occurrence data for Common Pink lantana (lineage A). Also plotted are collection coordinates of pink‐flowered plants used for host specificity testing of the biological control agent, the rust fungus *Prospodium tuberculatum*, together with susceptibility of each host plant to the rust (data from Thomas et al. 2006). Most of the susceptible pink‐flowered plants (13 out of 14) were sourced from localities where habitat was predicted to be moderately to highly suitable for Common Pink (> ~0.25), while most of the non‐susceptible pink‐flowered plants (four out of six) were collected from habitat predicted to be less suitable (< ~0.25).

Due to the infrequent and geographically restricted distribution of most genetic lineages (particularly C, E, G) the number of occurrence records available for modelling these was very low (Table 1, Figure S2). Thus, the accuracy of the predictions for these lineages is expected to be low compared with predictions for larger groups (e.g., lineage A, Figure 6; all samples combined, Figure S6); all results are nevertheless shown in Figure S6. The predicted suitable habitat of most individual genetic lineages largely coincided with their sampled distributions within the current known distribution of lantana in Australia, except for lineages D, F and G, for which we predicted more extensive suitable habitat than they currently occupy (Figure S6). However, since the occurrence data available for individual genetic lineages was limited, the accuracy of these predictions should be interpreted with caution. Combining occurrence data for all genetic lineages resulted in predicted suitable habitat largely coinciding with the area already occupied by lantana in Australia (Figure S6).

## 4 Discussion

### 4.1 Australian Lantana Comprises Multiple, Divergent Genetic Lineages

At least seven distinct, monophyletic genetic clusters (i.e., lineages) were found to make up the invasive lantana complex in Australia. Flower colour morphotype was consistent within genetic lineages, but failed to diagnose them, for example, most pink‐flowered samples (59%) belonged to lineage A, but 20% belonged to lineage F, and 21% were unclustered (Table 1). High inter‐lineage genetic divergence, even in sympatry (Figure 4), suggests limited gene flow between lineages, supporting the hypothesis of an invasion history involving multiple independent lineages warranting formal taxonomic recognition.

Lineages A and B, the two most widespread and abundant in Australia, respectively extended 2250 and 1870 km along the eastern coast, spanning ~19° and ~16° of latitude (Figure S2). They exhibited the two most common morphotypes (pink and pink‐edged red, respectively). Because our sampling was designed to represent all flower colour morphotypes as evenly as possible, the proportion of lineages A and B in our dataset (~40%) was an under‐representation of their abundance in the landscape; we suggest that they comprise the vast majority of all invasive lantana in Australia (supported by our field observations and consistent with Day, Broughton, and Hannan‐Jones 2003; Smith and Smith 1982). The results of species distribution modelling for these two lineages suggest that they currently occupy most of their potential Australian distribution (Figures 6, S2 and S6). Although our predictions were necessarily based on a limited number of records for individual genetic lineages, the prediction of suitable habitat for all samples combined shows a similar extent to the individual predictions obtained for lineages A and B (Figure S6).

Genome size estimates in lineages A and B were comparable with published estimates for tetraploid *Lantana* Sect. *Lantana*; small differences between estimates may reflect variation in DNA content and arrangement, or experimental error. Because our genome size estimates were only for a small subset of individuals of these two genetic lineages, further work is needed to build a comprehensive understanding of ploidy variation in Australian lantana. However, our preliminary finding that most of the invasive lantana in Australia is likely to be tetraploid supports previous reports based on unpublished data (Everist 1981; Smith and Smith 1982) and is consistent with findings from India (Praveen et al. 2025). Thus, ploidy variation is unlikely to act as a reproductive barrier between the main lineages of Australian lantana.

Genetic differentiation between lineages A and B was high (*F*_ST_~1; Figures 3 and 4); consistent with this, hybrids between them were scarce (only found at two sites; Figure S5, Table S5). Genetic diversity (i.e., allele frequency variation and observed heterozygosity) within both lineages A and B was remarkably low (Figures 4 and S3; Tables S3 and S4). Populations within lineages were not detectably differentiated, even across large geographic distances; for example, *F*_ST_ between lineage A populations from Diwan (Daintree; north Qld) and Norah Head (central NSW) was ~0, despite being separated by > 2000 km. Observed heterozygosity was distinctly low in lineage F as well as in lineages A and B (although C, D and G had higher observed heterozygosity). Low observed heterozygosity and lack of isolation by distance are consistent with recent range expansion following a genetic bottleneck involving inbreeding (e.g., self‐fertilisation), and mating systems involving preferential self‐fertilisation have been invoked to explain similar patterns in invasive lantana in India (Praveen et al. 2025). However, we note that these findings may also partly reflect SNP‐calling biases in polyploid, multi‐species datasets. While our results indicated limited gene flow between lantana genetic lineages, we also showed that inter‐lineage hybridisation is possible (albeit rare), suggesting that genetic lineages may not be fully reproductively isolated. This study lays a foundation for further investigation into mating systems and demographic processes in individual genetic lineages, for example, to characterise the relative strength and mechanism(s) of putative reproductive barriers between them.

### 4.2 Multiple Invasive Lantana Taxa Can Be Recognised in Australia

Our results did not support the concept of treating Australian lantana as a single natural taxon or hybrid swarm (i.e., individuals freely interbreeding regardless of morphotype, resulting in population genetic structure aligning with expectations under isolation by distance; Figure 1). Instead, our findings aligned best with the taxonomic treatment of Smith and Smith (1982), who described 29 taxa (varieties). The seven distinct genetic lineages we identified (A—G) were clearly aligned with seven of these varieties (Table 1). Further work is needed to understand the remaining 22 varieties in the context of our findings, for example, whether they were insufficiently sampled or otherwise failed to meet the criteria we used for identifying genetic lineages (discussed further below), or whether they are still currently extant. Additionally, we acknowledge that Smith and Smith's (1982) concepts have been historically under‐utilised due to the difficulty in applying them to identify individual specimens (see Methods). Nevertheless, our findings identified this treatment as the best‐supported taxonomic framework currently available for Australia, and we hereafter refer to the seven Australian genetic lineages identified in this study by their corresponding varietal names sensu Smith and Smith (1982); (Table 1).

Separate to these seven varieties, many of the Australian lantana individuals sampled here (38%) could not be assigned to a genetic lineage using the HDBSCAN clustering approach (i.e., were unclustered). Unclustered samples occurred throughout the full geographic area sampled (Figure S2), encompassed all morphotypes (Table 1, Figure 3), and varied widely in genetic diversity (Figure S3). Some were inter‐lineage hybrids, while others likely represented genetic lineages undetected due to insufficient sampling (< 10 samples, see Methods). For example, hybrids between lineages A and B (Figure S5, Table S5) were unclustered, as were some samples from single sites that comprised unique ancestral populations at *K* = 11 (Figure 3e). We note that the high proportion of unclustered samples in our dataset is more likely to reflect a deliberate bias in our sampling than the true distribution of genetic lineages in the landscape. Because we attempted to represent the full extent of lantana morphological diversity as evenly as possible, we over‐represented morphological outliers compared with their abundance relative to the common morphotypes in the wild. Thus, while Australian lantana includes some populations consisting of highly localised genetic lineages and/or inter‐lineage hybrids, at the national scale, the complex can be broadly understood as comprising seven main invasive lineages, of which two are dominant (i.e., lineages A and B; Common Pink and Common Pink‐Edged Red).

To produce a broadly applicable taxonomic treatment for Australia, further work is needed to review and revise the varietal concepts of Smith and Smith (1982), as well as to place genetically unclustered populations in this taxonomic framework. Such a revision would need to compile updated information on the monophyly, distribution, distinguishing traits, toxicity and biological control agent susceptibility of each variety. The complexity of invasive lantana as illustrated by this study attests to the challenges involved in its taxonomic treatment, but our findings offer a new way forward to solve this longstanding problem.

### 4.3 The Provenance and Evolutionary History of Invasive Lantana Remain Cryptic

Our analyses included non‐hybrid specimens of nine native‐range *Lantana* sect. *Lantana* species, but none of the seven main Australian lineages were closely related to them. Most native‐range samples fell into two clades which included only a few Australian unclustered samples (Figure 5). Three Australian lineages were related to single, putatively hybrid, native‐range samples from Brazil (lineage A; Common Pink) and Mexico (lineages B, F; Common Pink‐Edged Red, Townsville Red‐Centred Pink). No native‐range sister lineage was identified for the remaining four Australian lineages. Our inability to identify the native‐range provenance of Australian lantana may be due to limited sampling of the Americas: *Lantana* sect. *Lantana* comprises 20 species (some rare or even extinct in the wild; Sanders 2006, 2012) distributed from the southern USA to northern Argentina, but only nine were included in our analyses. It may also be due to the confounding effect of Australian genetic lineages having been derived from garden‐origin hybrids; Smith and Smith (1982) expressed the opinion that Australian lantana was distinct from naturally‐occurring species and invoked this reason to explain it. The introduction, naturalisation and spread of multiple hybrid lantana cultivars, possibly with further spontaneous introgression taking place outside of cultivation, would make inferring the ancestral range of present‐day invasive populations difficult, if not impossible.

It is unknown how hybridisation and artificial selection in cultivation may have contributed to the genetic makeup of present‐day invasive lantana populations. Howard (1969) compiled a comprehensive list of > 600 cultivar names going back > 200 years and stated that ‘Any attempt to apply the descriptions recorded to specific plants today would be futile’, and that ‘The vast majority of the names in the following list are not offered in commerce today’. Additional records are available for some modern‐day cultivars (e.g., Czarnecki et al. 2014; Deng et al. 2017) but further work is needed to reveal how they might be related to invasive populations. The idea that hybridisation could produce novel plant lineages with the adaptive traits necessary to invade novel environments has been of great interest in evolution and ecology (e.g., Anderson and Stebbins 1954; Pfennig et al. 2016; Porretta and Canestrelli 2023); invasive lantana might provide a new and potentially promising system for experimental research on this topic.

For reasons discussed above, identification of native‐range provenances of Australian lantana lineages should be treated with caution. However, we hypothesise based on our preliminary results that Common Pink (lineage A) was derived from plants native to Brazil, while progenitor(s) of Common Pink‐edged Red (lineage B) and Townsville Red‐centred Pink (lineage F) were likely native to Mexico. Testing this hypothesis will require further investigation with potentially important implications for improving lantana biological control (discussed below). We focused on characterising genetic diversity and structure in Australian invasive lantana, rather than on comprehensive phylogenetic reconstruction of *Lantana* sect. *Lantana*, limiting the broader evolutionary implications that can be drawn from this study. Ideally, a global study with comprehensive sampling and using a combination of phylogenomic, population genomic and morphological data should be undertaken to identify invasive lineages worldwide, and clarify their relationships to native species. This would also lay the necessary foundation for a globally comprehensive monograph and taxonomic revision of *Lantana* sect. *Lantana*.

### 4.4 Implications for Biological Control

Our support for recognising multiple, genetically distinct lantana taxa in Australia has clear management implications. While the effectiveness of chemical and mechanical control does not depend on precise identification of lantana populations, other management approaches require a more nuanced, taxon‐specific strategy (Pyšek et al. 2013). For example, biological control and assessments of weed impact and risk at regional scales must consider each genetic lineage and its associated specific agents, toxicity and bioclimatic requirements to optimise management outcomes (Day, Wiley, et al. 2003). The concept of five flower‐colour morphotypes employed by Australian biological control researchers to classify lantana failed to distinguish between different genetic lineages of potentially different provenance. Thus, identifying host background using flower colour alone might result in prospecting for biological control agents in the wrong locations, and/or deploying agents on the wrong hosts. A taxonomic revision is needed to provide an accurate and applicable framework for identifying host variation in lantana for the purposes of biological control.

Variation in host preferences among Australian lantana biological control agents supports our findings and underscores the value of accounting for the taxonomic identity of lantana populations to improve control success. For example, host‐specificity tests found that the leaf rust *Prospodium tuberculatum* (Speg.) Arthur (Pucciniales) established predominantly on pink‐flowered plants, but also that ~25% of the pink‐flowered plants tested were immune to it (Thomas et al. 2006). We were unable to sample the original host plants tested, but based on Thomas et al.'s (2006) collection data, most susceptible hosts came from sites matching our predicted distribution of Common Pink (lineage A), while most non‐susceptible pink‐flowered plants came from outside this range (Figure 6), consistent with *P. tuberculatum* being specific to Common Pink. Further, *P. tuberculatum* introduced to Australia was collected in Brazil (Thomas et al. 2006), consistent with our hypothesised Brazilian ancestry for Common Pink. The flower‐galling mite *Aceria lantanae* Cook (Eriophyidae) provides another example illustrating the taxonomic distinction between different pink‐flowered genetic lineages. Both of these biological control examples are summarised in Table S6.

Due to the presence of multiple distinct and divergent genetic lineages within the invasive lantana complex in Australia, it is unlikely that any one biological control agent will provide effective control. Even so, biological control has an important role in integrated management of lantana (Day, Wiley, et al. 2003). To maximise effectiveness, different suites of agents will need to be tailored to different areas invaded by lantana according to which host genetic lineages are dominant. The bioclimatic requirements of prospective control agents may also limit their establishment and effectiveness, particularly in instances where invasive lantana has colonised habitats unsuitable for the agent (e.g., *Prospodium tuberculatum* is poorly known from the southern NSW section of the distribution of Common Pink lantana; Day, Broughton, and Hannan‐Jones 2003; GBIF.org 2026a). Future biological control research in Australia should take into account the bioclimatic requirements of host lineages together with those of control agents to maximise success.

We explored the prediction of suitable habitat for each of the seven main genetic lineages of lantana in Australia, using the limited number of occurrence records available from our study. We note that MaxEnt performs better with few records relative to other methods (Pearson et al. 2007) and our use of many environmental predictors should have improved accuracy in this case (Merow et al. 2013). However, we also note that our input data did not meet optimal specifications (Elith et al. 2011; Hui 2022) and that our results should thus be treated with caution. For the two dominant genetic lineages (Common Pink and Common Pink‐Edged Red, lineages A and B) as well as for all lantana samples combined (i.e., all seven lineages plus unclustered samples), we predicted that their suitable habitat in Australia largely matches their current distribution, that is, the present‐day risk of invading new areas is low. For the less widespread genetic lineages, we predicted either very limited or very extensive suitable habitat (Figure S6), but these results were based on very few records and are thus less likely to be accurate. Prior predictions of current and future suitable habitat for lantana in Australia have varied greatly depending on the methods used (Adhikari et al. 2024; Goncalves et al. 2014; Taylor et al. 2012; Taylor and Kumar 2013; van Oosterhout 2004). These studies all used the broad concept of “ *Lantana camara* ” for the species complex. The name *Lantana camara* has been applied to a large number of plants around the world (GBIF.org 2026b), encompassing invasive, cultivated and native‐range individuals. We hypothesise that the use of this broad concept misses a great deal of taxonomic variation, and might thus affect the accuracy of predictions (e.g., Mori et al. 2019), but this can only be further investigated after a better understanding of *Lantana* sect. *Lantana* taxonomy is achieved on a global scale.

In summary, our findings provide a critical foundation for designing customised biological control strategies, and highlight the importance of population genetic approaches to supporting biological control of weeds (reviewed by Gaskin 2024). Further research into lantana biological control on a global scale might use population genomic data to identify and compare genetic lineages present in other countries with those present in Australia, to predict which agents used in one country (Table S7) might be successful in others based on the presence of suitable hosts. Finally, by providing testable hypotheses of the native provenance of different genetic lineages of invasive lantana, we set the stage for further prospecting for new, more effective biological control agents.

### 4.5 Conclusion

While the evolutionary history and taxonomy of Australian invasive lantana is complicated, we have made progress toward clarifying longstanding issues. We identified distinct genetic lineages corresponding to taxonomic varieties, likely descended from multiple progenitor species from different parts of the native range. The genetic lineages corresponding with the varieties Common Pink and Common Pink‐edged Red are dominant in Australia. Genome‐wide marker (SNP) data across the full invaded range was key to detecting genetic structure and lineages in invasive lantana in Australia, demonstrating the applicability of population genomics for resolving invasive species complexes. This study represents a starting point towards better understanding the taxonomy of invasive lantana on a global scale. In addition to taxonomic revision, future work should integrate comparative genomic analyses of invasive lantana across global regions, particularly where biological control programs are in place (e.g., South Africa and Hawaii), which will enable strategic sharing of knowledge and resources (e.g., existing biological control agents or the identification of new ones) when the same lantana lineages have invaded multiple regions. The inclusion of broader and more comprehensive sampling from the native range in these analyses, together with the assembly of reference genome sequences, should unlock additional insights into the origins of invasive lineages and facilitate investigation into the processes of adaptation and diversification. At a local scale, cross‐pollination studies could shed light on reproductive barriers between lineages, while host‐choice and no‐choice experiments would test lineage‐specificity in prospective biological control agents.

## Funding

This study was funded by the Australian Government, Department of Agriculture, Fisheries and Forestry (activity ID: 4‐FY9KQZ3).

## Conflicts of Interest

The authors declare no conflicts of interest.

## Supporting information


**Data S1:** SDM response curves.

**Table S1:** Details and metadata for samples used in this study.
**Table S2:** Species distribution model performance statistics for all genetic lineages of Australian lantana populations.
**Table S3:** Summary statistics of individual heterozygosity and inbreeding for each genetic lineage.
**Table S4:** Adjusted *p*‐values from post hoc pairwise Wilcoxon tests (Bonferroni correction) comparing individual heterozygosity (Ho) between genetic lineages.
**Table S5:** NewHybrids assignment probabilities for putative hybrids.
**Table S6:** Summary of biological control agent preferences for pink‐flowered host varieties for two agents with inconsistent establishment on pink‐flowered plants.
**Table S7:** Biological control agents deployed in only one country.
**Figure S1:** Genome size estimates using flow cytometry.
**Figure S2:** Geographic distribution of lantana samples assigned to genetic lineages based on population analysis.
**Figure S3:** Individual observed heterozygosity (Ho) at loci with a minimum minor allele frequency (MAF) > 2%.
**Figure S4:** Extended LEA snmf results for K = 3, 5, 7, 9 and 11 on eastern Australian lantana (grouped by genetic lineages); cross entropy plotted for different values of K.
**Figure S5:** Visualisation of genetic evidence for hybridization between Common Pink and Common Pink‐Edged Red lantana (lineages A and B), based on 33 individuals from four sites.
**Figure S6:** Habitat suitability predictions for (a)–(g) each of seven lantana genetic lineages identified; (h) all individuals sampled from invasive populations as part of this study.

**Table S1:** eva70251‐sup‐0003‐TableS1@Table S1 sample metadata.csv.

**Table S7:** eva70251‐sup‐0004‐TableS7@Table S7 agents in only one country.xlsx.

## Data Availability

The raw genotype matrices analysed in this study are available via Zenodo (https://doi.org/10.5281/zenodo.17224144) and the metadata are included as Supporting Information to the paper. Climate data (9 s resolution) are available from the CSIRO Data Access Portal at https://doi.org/10.25919/5dce30cad79a8 (Bio4‐Bio32), https://doi.org/10.4225/08/5afa9f7d1a552 (EAA, EPI, WDI and WDX) and https://doi.org/10.4225/08/5b285fd14991f (CLY, SND, NTO, PTO, BDW and TWI3S).
