# Supplementary material for: Tangled Evolutionary History: Genetically Divergent Taxa and Hybrids Characterise Lantana Invasions in Australia
Source: Evol Appl. 2026 May 20;19(5):e70251. doi: 10.1111/eva.70251 (PMC13239396; doi:10.1111/eva.70251)

Supplementary Materials

Here we show the response curves of the top four environmental predictors based on permutation test in MaxEnt for all clusters and the analysis considering all lantana records:

# Cluster A


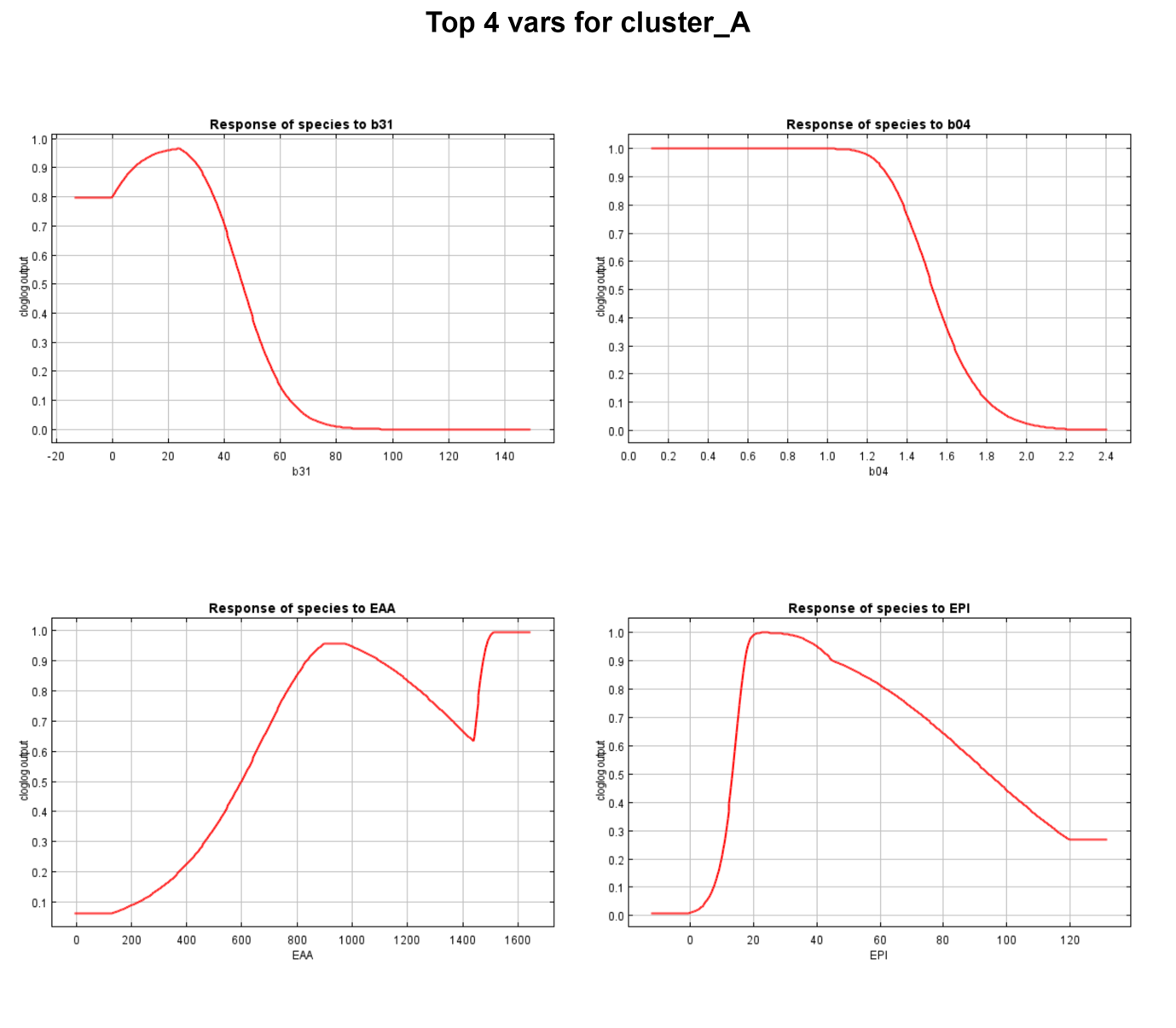


# Cluster B


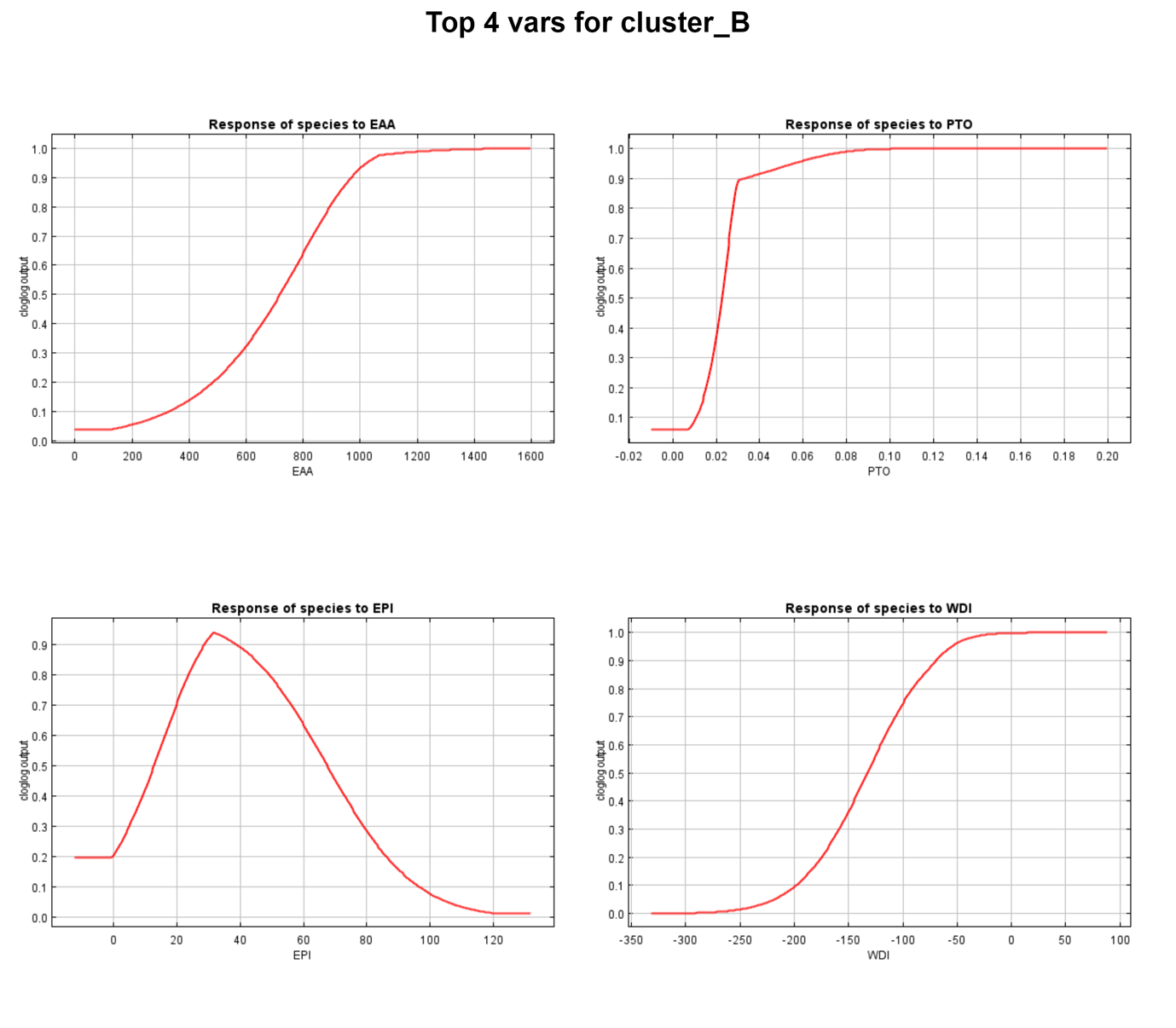


# Cluster C


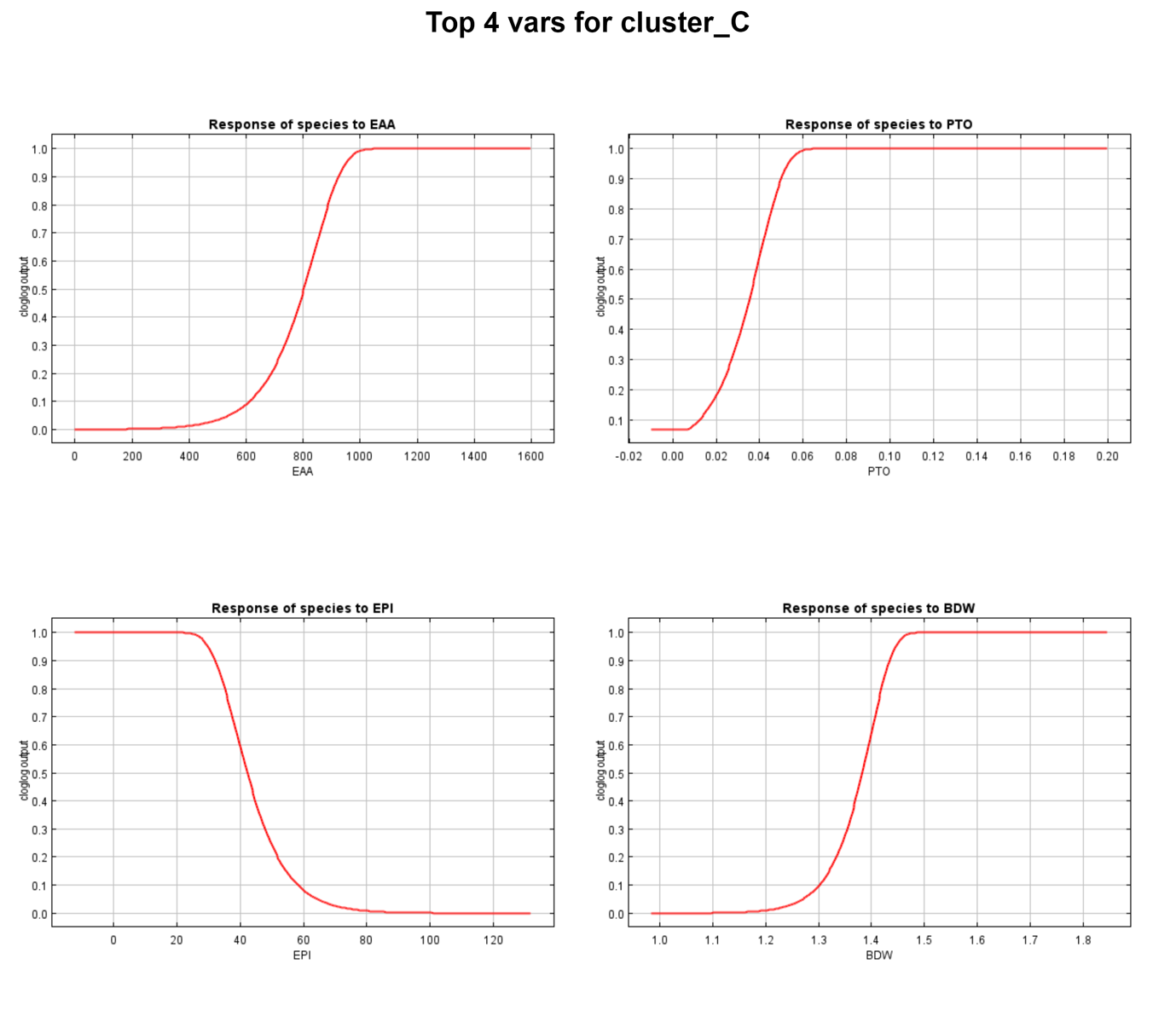


## Cluster D

#
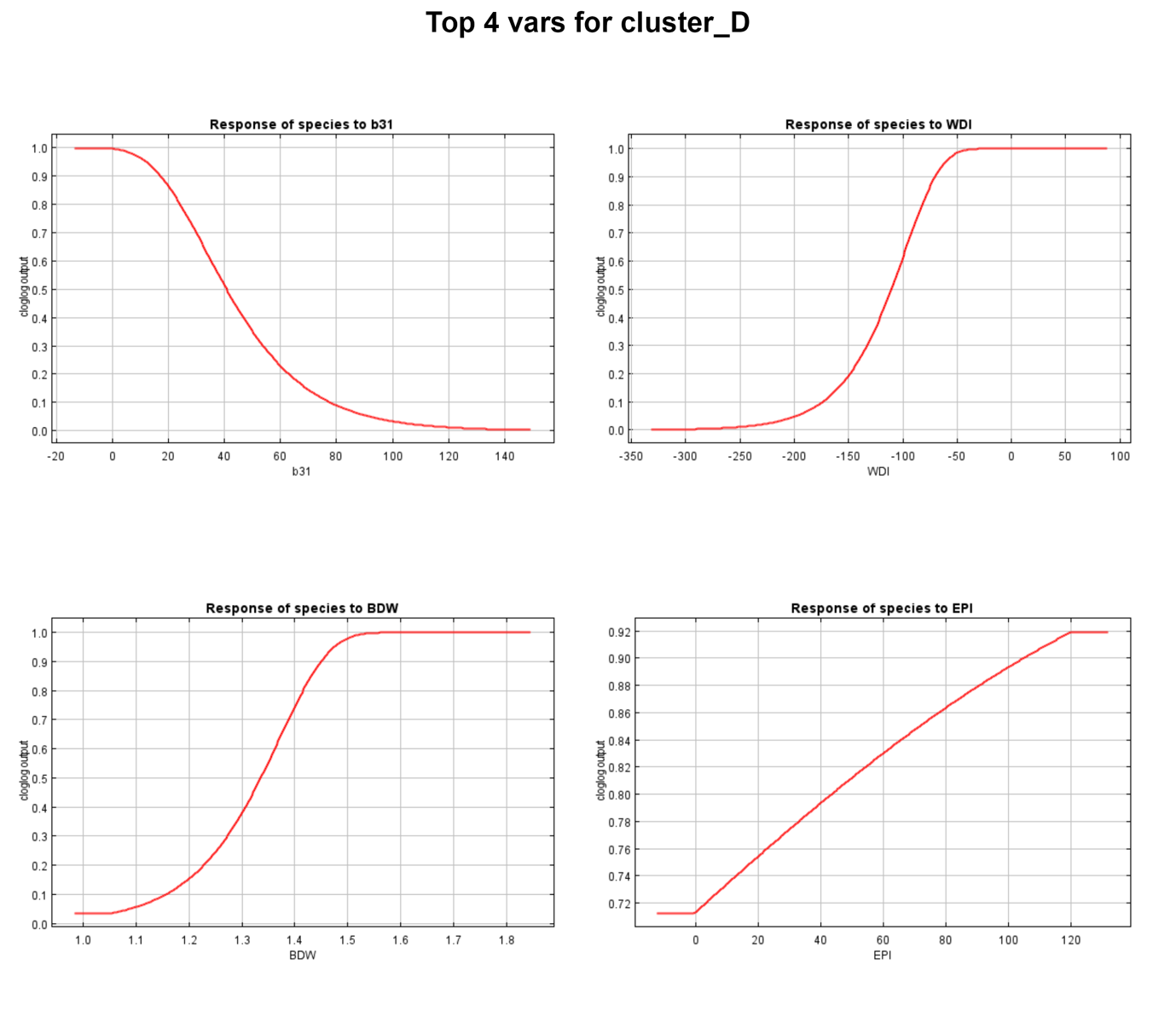


# Cluster E


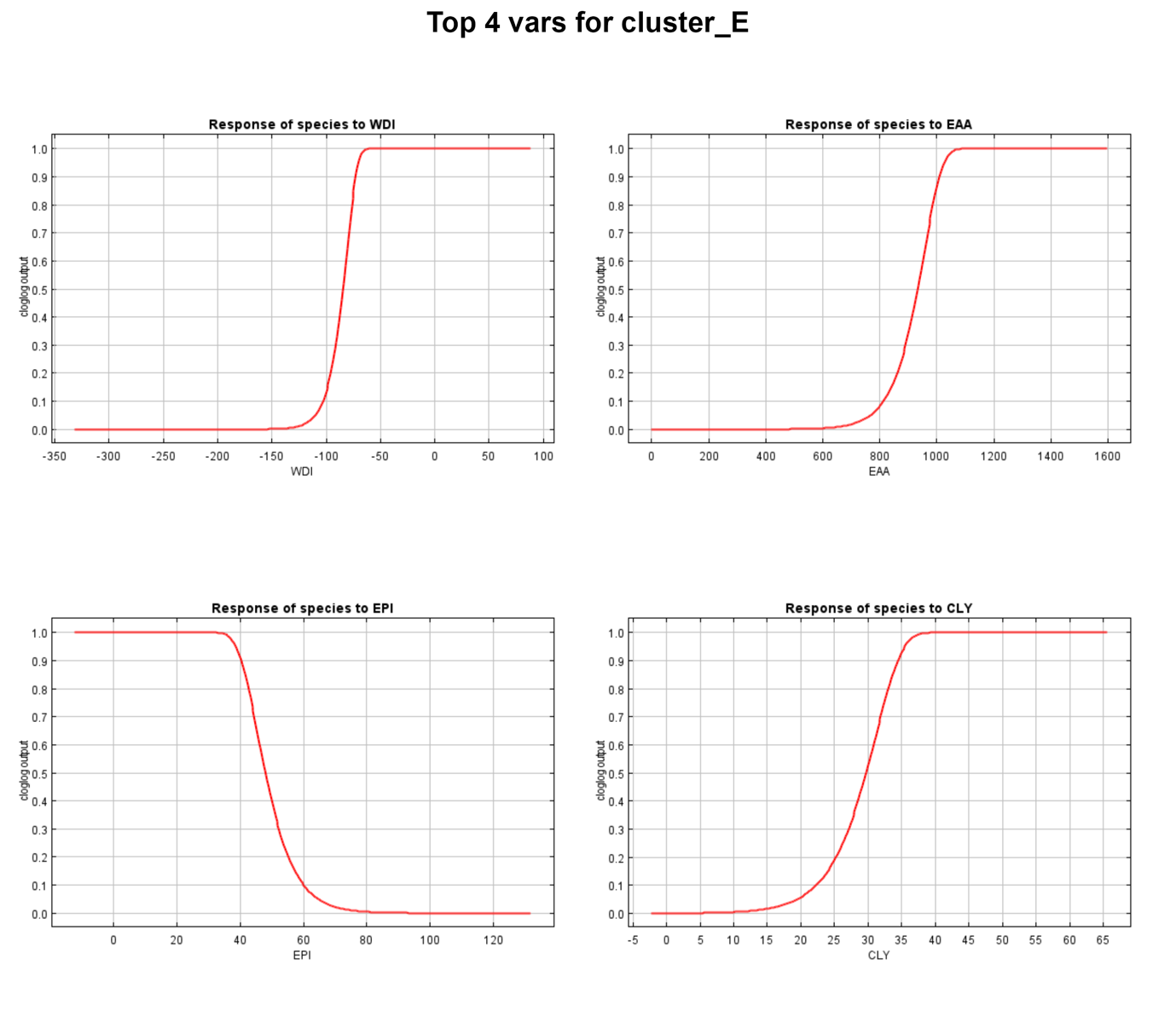


# Cluster F


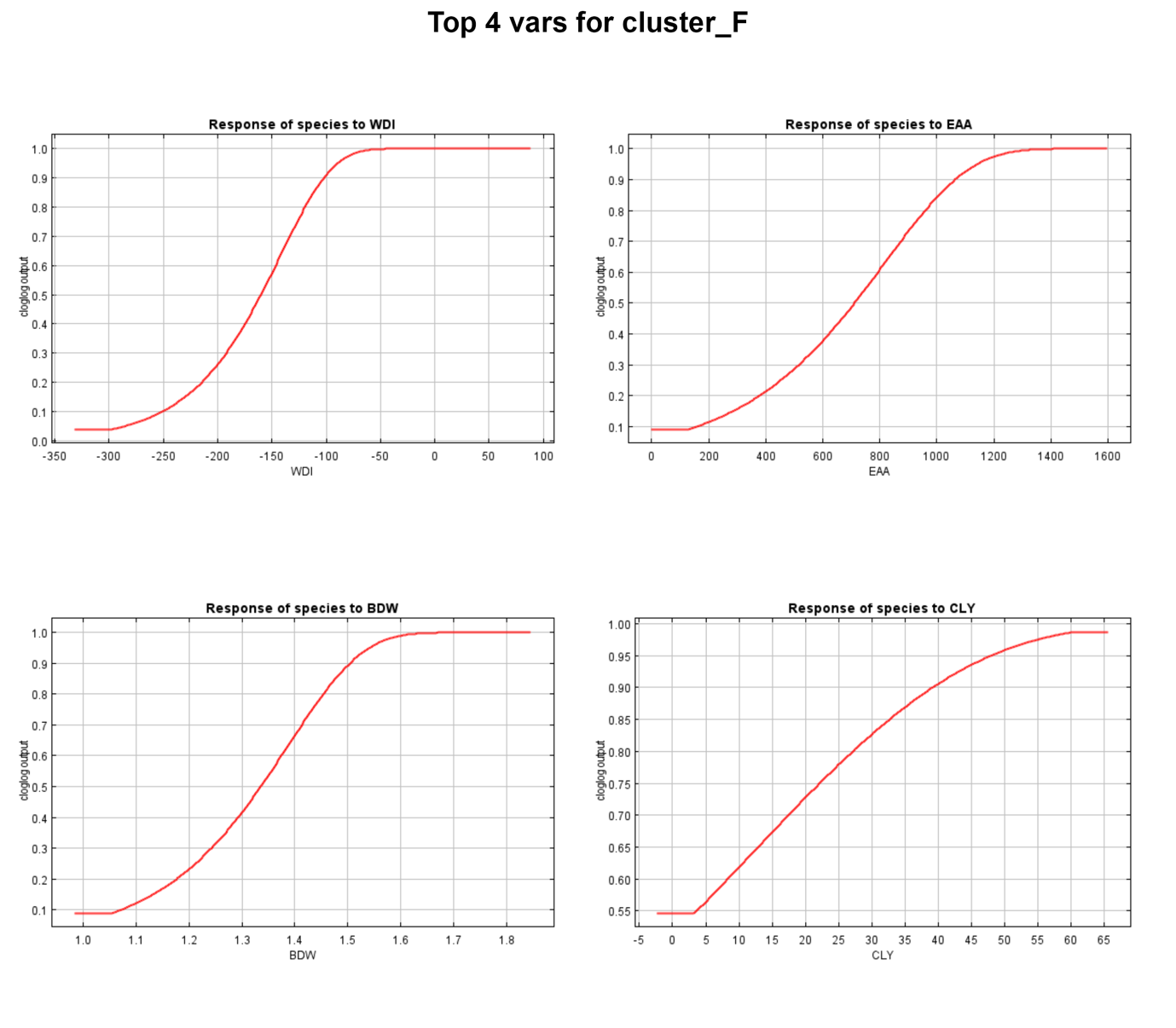


# Cluster G


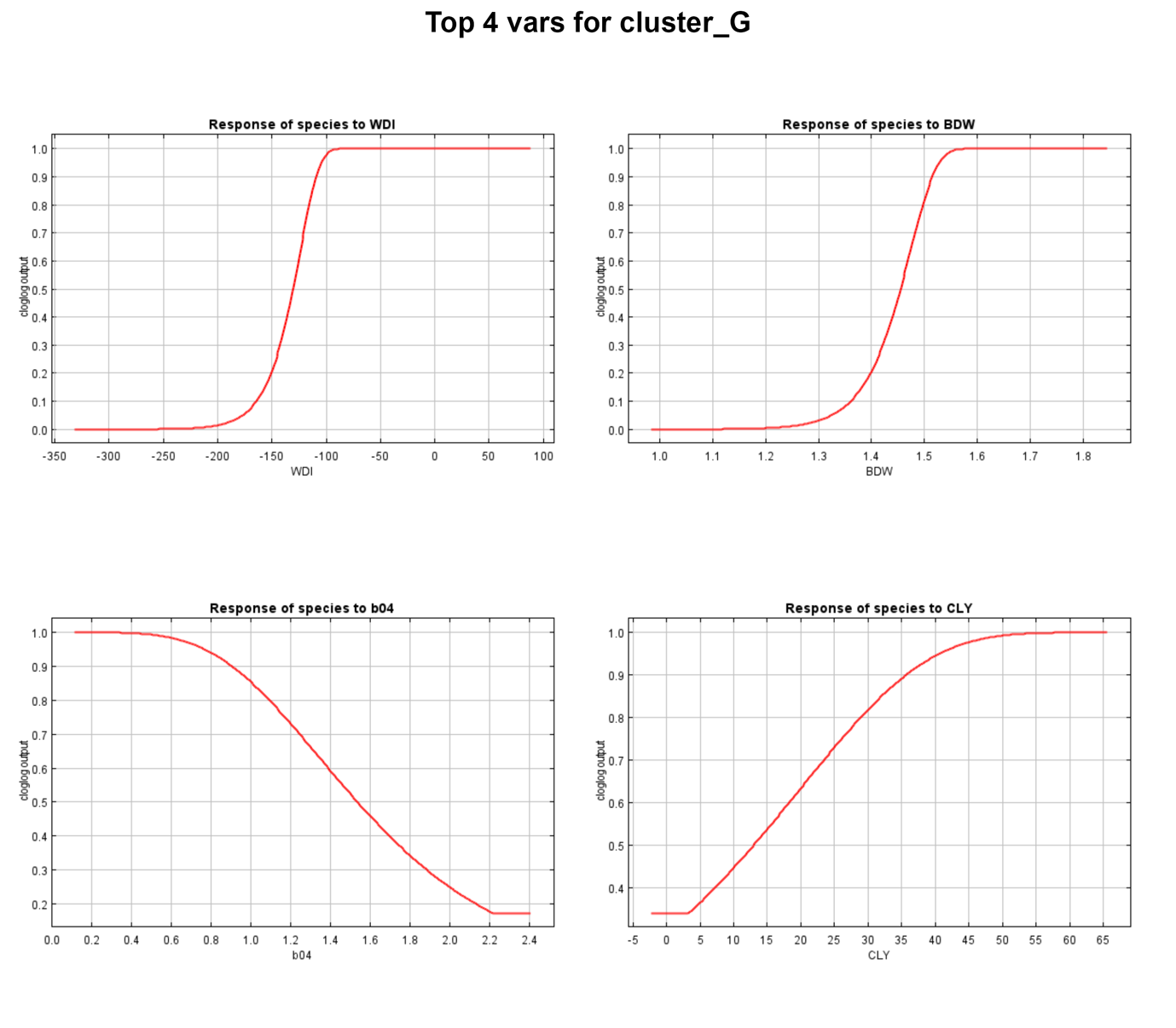


# Lantana – all records


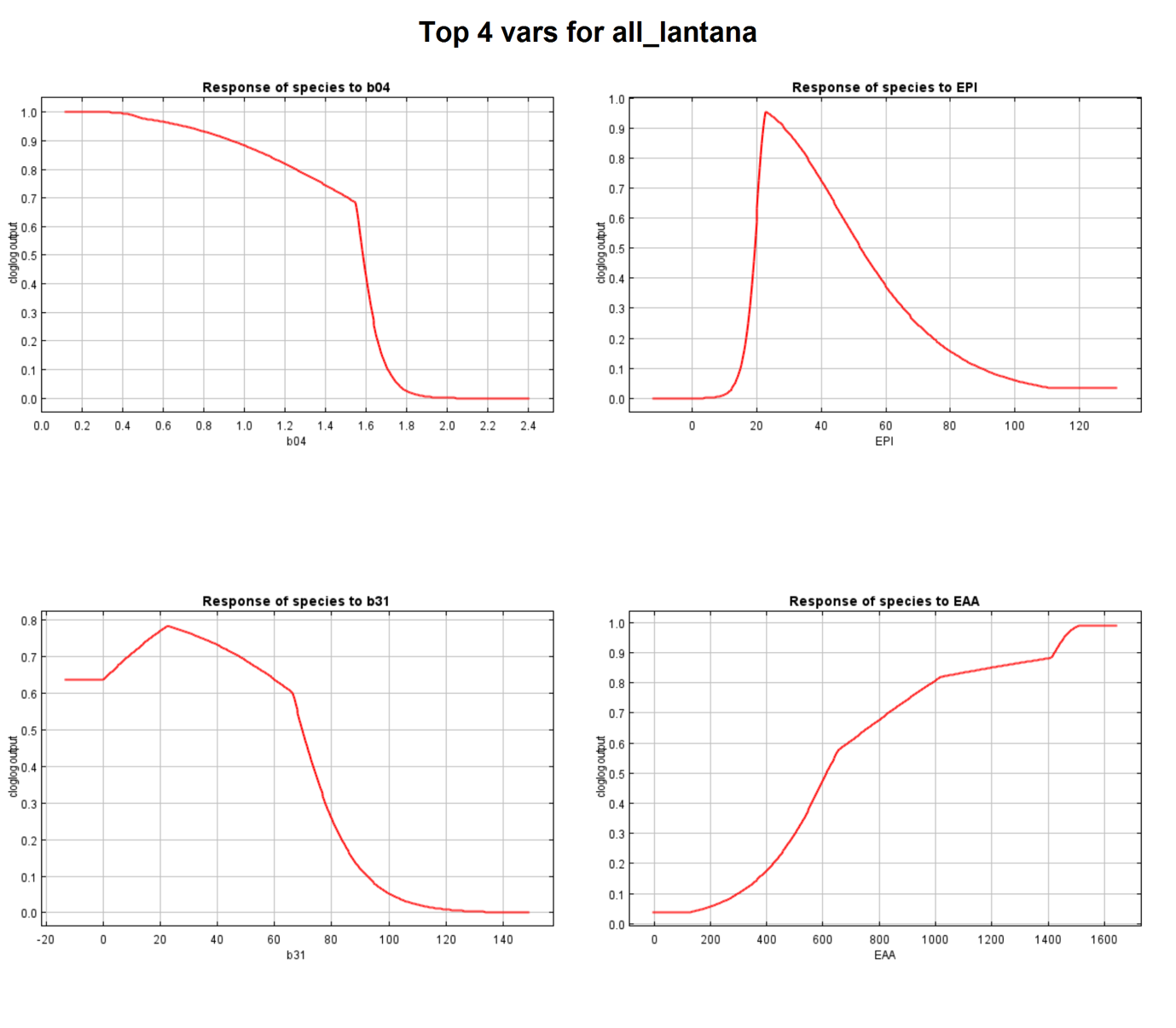

Supplement: Supplementary file 1 — Data S1: SDM response curves. [file EVA-19-e70251-s002.docx]
