# Supplementary material for: Tangled Evolutionary History: Genetically Divergent Taxa and Hybrids Characterise Lantana Invasions in Australia
Source: Evol Appl. 2026 May 20;19(5):e70251. doi: 10.1111/eva.70251 (PMC13239396; doi:10.1111/eva.70251)
Supplement: Supplementary file 2 — Table S1: Details and metadata for samples used in this study. Table S2: Species distribution model performance statistics for all genetic lineages of Australian lantana populations. Table S3: Summary statistics of individual heterozygosity and inbreeding for each genetic lineage. Table S4: Adjusted p‐values from post hoc pairwise Wilcoxon tests (Bonferroni correction) comparing individual heterozygosity (Ho) between genetic lineages. Table S5: NewHybrids assignment probabilities for putative hybrids. Table S6: Summary of biological control agent preferences for pink‐flowered host varieties for two agents with inconsistent establishment on pink‐flowered plants. Table S7: Biological control agents deployed in only one country. Figure S1: Genome size estimates using flow cytometry. Figure S2: Geographic distribution of lantana samples assigned to genetic lineages based on population analysis. Figure S3: Individual observed heterozygosity (Ho) at loci with a minimum minor allele frequency (MAF) > 2%. Figure S4: Extended LEA snmf results for K = 3, 5, 7, 9 and 11 on eastern Australian lantana (grouped by genetic lineages); cross entropy plotted for different values of K. Figure S5: Visualisation of genetic evidence for hybridization between Common Pink and Common Pink‐Edged Red lantana (lineages A and B), based on 33 individuals from four sites. Figure S6: Habitat suitability predictions for (a)–(g) each of seven lantana genetic lineages identified; (h) all individuals sampled from invasive populations as part of this study. [file EVA-19-e70251-s001.docx]

# SUPPLEMENTARY TABLES AND FIGURES

#### Table S1. Details and metadata for samples used in this study

[attached separately]

#### Table S2. Species distribution model performance statistics for all genetic lineages of Australian lantana populations

Area under the Received Operating Characteristic curve (AUC), maximum of Cohen’s Kappa (K) threshold, and Maximum training sensitivity and specificity logistic threshold (MTSS). We also show the two most important environmental predictors for each genetic lineage’s SDM found by MaxEnt in the column called ‘Environmental predictors’. B04: temperature seasonality (°C); B31: Moisture index seasonality (C of V); EAA: Annual total actual evapotranspiration; PTO: Total Phosphorus; WDI: Minimum monthly atmospheric water deficit (precipitation - potential evaporation).

| ***Genetic lineage*** | ***AUC*** | ***Kappa*** | ***MTSS*** | ***Environmental predictors*** |
| --- | --- | --- | --- | --- |
| A | 0.99 | 0.71 | 0.21 | *B31, EAA* |
| B | 0.99 | 0.86 | 0.07 | *WDI, EAA* |
| C | 0.99 | 0.94 | 0.64 | *EAA, PTO* |
| D | 0.98 | 0.85 | 0.47 | *WDI, B31* |
| E | 0.99 | 0.87 | 0.87 | *EAA, B31* |
| F | 0.97 | 0.90 | 0.36 | *B04, EAA* |
| G | 0.98 | 0.88 | 0.64 | *B04, WDI* |

#### Table S3. Summary statistics of individual heterozygosity and inbreeding for each genetic lineage

Median observed heterozygosity (*H_O_*) and inbreeding coefficient (*F_IS_*) of individuals within each lineage, with standard deviation (SD) shown. Sample size (*n*) indicates the number of individuals per lineage. Data presented here is the same as in Fig. S3.

| Lineage | *n* | *H_O_* (median ± SD) | *F_IS_* (median ± SD) |
| --- | --- | --- | --- |
| A | 115 | 0.015 ± 0.004 | 0.923 ± 0.022 |
| B | 82 | 0.003 ± 0.003 | 0.986 ± 0.017 |
| C | 12 | 0.153 ± 0.02 | 0.198 ± 0.103 |
| D | 46 | 0.12 ± 0.022 | 0.371 ± 0.114 |
| E | 13 | 0.151 ± 0.034 | 0.208 ± 0.177 |
| F | 39 | 0.019 ± 0.008 | 0.9 ± 0.042 |
| G | 11 | 0.06 ± 0.045 | 0.688 ± 0.234 |
| Unclustered Australian | 206 | 0.108 ± 0.056 | 0.434 ± 0.294 |
| Native range | 128 | 0.026 ± 0.031 | 0.863 ± 0.165 |

#### Table S4. Adjusted p-values from post hoc pairwise Wilcoxon tests (Bonferroni correction) comparing individual heterozygosity (H_O_) between genetic lineages

The Kruskal-Wallis test showed a significant difference in *H_O_* among lineages (χ² = 403.38, df = 8, *p* < 2.2e-16). Values represent adjusted p-values for pairwise comparisons, with significant differences (*p* < 0.05) highlighted.

|  | A | B | C | D | E | F | G | Unclustered Australian |
| --- | --- | --- | --- | --- | --- | --- | --- | --- |
| B | 0.000 |  |  |  |  |  |  |  |
| C | 0.000 | 0.000 |  |  |  |  |  |  |
| D | 0.000 | 0.000 | 0.001 |  |  |  |  |  |
| E | 0.000 | 0.000 | 1.000 | 0.022 |  |  |  |  |
| F | 0.260 | 0.000 | 0.000 | 0.000 | 0.000 |  |  |  |
| G | 0.000 | 0.000 | 0.020 | 1.000 | 0.055 | 0.000 |  |  |
| Unclustered Australian | 0.000 | 0.000 | 0.016 | 1.000 | 0.071 | 0.000 | 1.000 |  |
| Native range | 0.000 | 0.000 | 0.000 | 0.000 | 0.000 | 0.029 | 0.011 | 0.000 |

#### Table S5. NewHybrids assignment probabilities for putative hybrids

Results for 33 lantana individuals from four sites (two with uniform flower colour and two with mixed/intermediate colours), testing putative hybridization between Common Pink and Common Pink–Edged Red lantana (lineages A and B). NewHybrids was run in an unsupervised mode. Values indicate the posterior probability of assignment to each class: parental populations (P0, P1), first- and second-generation hybrids (F1, F2), and backcrosses to each parent (F1×P0, F1×P1). Parental lineages (A or B) reflect groups identified from prior population structure analyses.

| **ID** | **Cluster** | **P0** | **P1** | **F1** | **F2** | **F1xP0** | **F1xP1** |
| --- | --- | --- | --- | --- | --- | --- | --- |
| NSW1158946 | B | 0 | 1 | 0 | 0 | 0 | 0 |
| NSW1158947 | B | 0 | 1 | 0 | 0 | 0 | 0 |
| NSW1158948 | B | 0 | 1 | 0 | 0 | 0 | 0 |
| NSW1158949 | B | 0 | 1 | 0 | 0 | 0 | 0 |
| NSW1158950 | B | 0 | 1 | 0 | 0 | 0 | 0 |
| NSW1158952 | B | 0 | 1 | 0 | 0 | 0 | 0 |
| NSW1159089 | B | 0 | 1 | 0 | 0 | 0 | 0 |
| NSW1159091 | B | 0 | 1 | 0 | 0 | 0 | 0 |
| NSW1159092 | B | 0 | 1 | 0 | 0 | 0 | 0 |
| NSW1159093 | B | 0 | 1 | 0 | 0 | 0 | 0 |
| NSW1159094 | B | 0 | 1 | 0 | 0 | 0 | 0 |
| NSW1159103 | B | 0 | 1 | 0 | 0 | 0 | 0 |
| NSW1159104 | B | 0 | 1 | 0 | 0 | 0 | 0 |
| NSW1159105 | B | 0 | 1 | 0 | 0 | 0 | 0 |
| NSW1159106 | B | 0 | 1 | 0 | 0 | 0 | 0 |
| NSW1159107 | B | 0 | 1 | 0 | 0 | 0 | 0 |
| NSW1159110 | B | 0 | 1 | 0 | 0 | 0 | 0 |
| NSW1159071 | A | 1 | 0 | 0 | 0 | 0 | 0 |
| NSW1159072 | A | 1 | 0 | 0 | 0 | 0 | 0 |
| NSW1159073 | A | 1 | 0 | 0 | 0 | 0 | 0 |
| NSW1159074 | A | 1 | 0 | 0 | 0 | 0 | 0 |
| NSW1159075 | A | 1 | 0 | 0 | 0 | 0 | 0 |
| NSW1159076 | A | 1 | 0 | 0 | 0 | 0 | 0 |
| NSW1159098 | A | 1 | 0 | 0 | 0 | 0 | 0 |
| NSW1159090 |  | 0 | 0 | 0 | 1 | 0 | 0 |
| NSW1159095 |  | 0 | 0 | 0 | 1 | 0 | 0 |
| NSW1159096 |  | 0 | 0 | 0 | 1 | 0 | 0 |
| NSW1159097 |  | 0 | 0 | 0 | 1 | 0 | 0 |
| NSW1159099 |  | 0 | 0 | 0 | 1 | 0 | 0 |
| NSW1159100 |  | 0 | 0 | 0 | 1 | 0 | 0 |
| NSW1159101 |  | 0 | 0 | 0 | 1 | 0 | 0 |
| NSW1159102 |  | 0 | 0 | 0 | 1 | 0 | 0 |
| NSW1159108 |  | 0 | 0 | 0 | 1 | 0 | 0 |

#### Table S6. Summary of biological control agent preferences for pink-flowered host varieties for two agents with inconsistent establishment on pink-flowered plants

Thomas et al. (2006) found that *Prospodium tuberculatum* preferred pink-flowered plants, but also that ~25% of the pink-flowered plants tested were not susceptible. Most susceptible hosts came from sites matching our predicted distribution of Common Pink (lineage A) while most non-susceptible pink-flowered plants came from outside this range, suggesting host-specificity of *P. tuberculatum* to Common Pink. This supports the hypothesized Brazilian ancestry of this variety, since *P. tuberculatum* introduced to Australia was collected in Brazil (Thomas et al., 2006).

| **Agent** | **Agent origin** | **Susceptible morphotypes** | **Proposed preferred host** | **Origin of nearest native-range relative of proposed host** |
| --- | --- | --- | --- | --- |
| *Prospodium tuberculatum* | Brazil | most pink, but not all pink | Common Pink (lineage A) | Brazil |
| *Aceria lantanae* | USA | not pink, except in northern Queensland | Townsville Red-Centred Pink (lineage F) | Mexico |

Similarly, *Aceria lantanae* has generally failed to establish on pink-flowered lantana, except in northern Queensland (Murree 2017; 2018) within the distribution of Townsville Red-centred Pink (lineage F). We therefore suggest Townsville Red-centred Pink is susceptible to *A. lantanae*, whereas Common Pink is not. The hypothesised provenance of the susceptible host (North America) again aligns with the agent’s introduction source (Mukwevho et al., 2017).

Murree, K. (2017). iNaturalist observation: https://www.inaturalist.org/observations/181871895. Accessed on 19 June 2025.

Murree, K. (2018). iNaturalist observation: https://www.inaturalist.org/observations/181868045. Accessed on 19 June 2025.

Mukwevho, L., Simelane, D., & Olckers, T. (2017). Host-plant variety and not climate determines the establishment and performance of Aceria lantanae (Eriophyidae), a biological control agent of Lantana camara in South Africa. Experimental & Applied Acarology, 71(2), 103–113.

#### Table S7. Biological control agents deployed in only one country

[attached separately]

#### Fig. S1. Genome size estimates


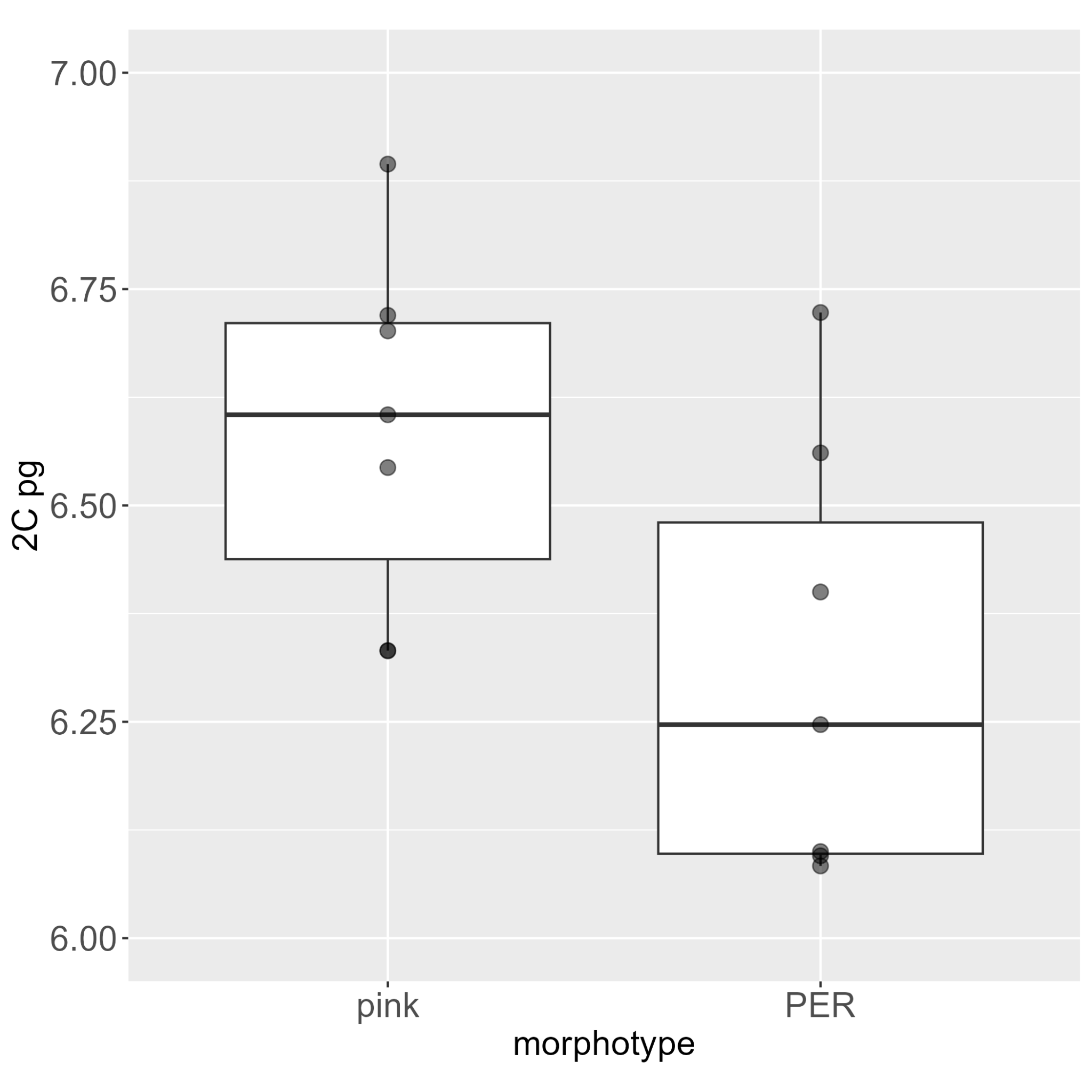
Estimates of genome size (DNA content) in picograms from flow cytometric analysis of nuclei extracted from 14 wild-sampled individuals of two lantana morphotypes (pink, pink-edged red).

#### Fig. S2. Genetic lineage maps


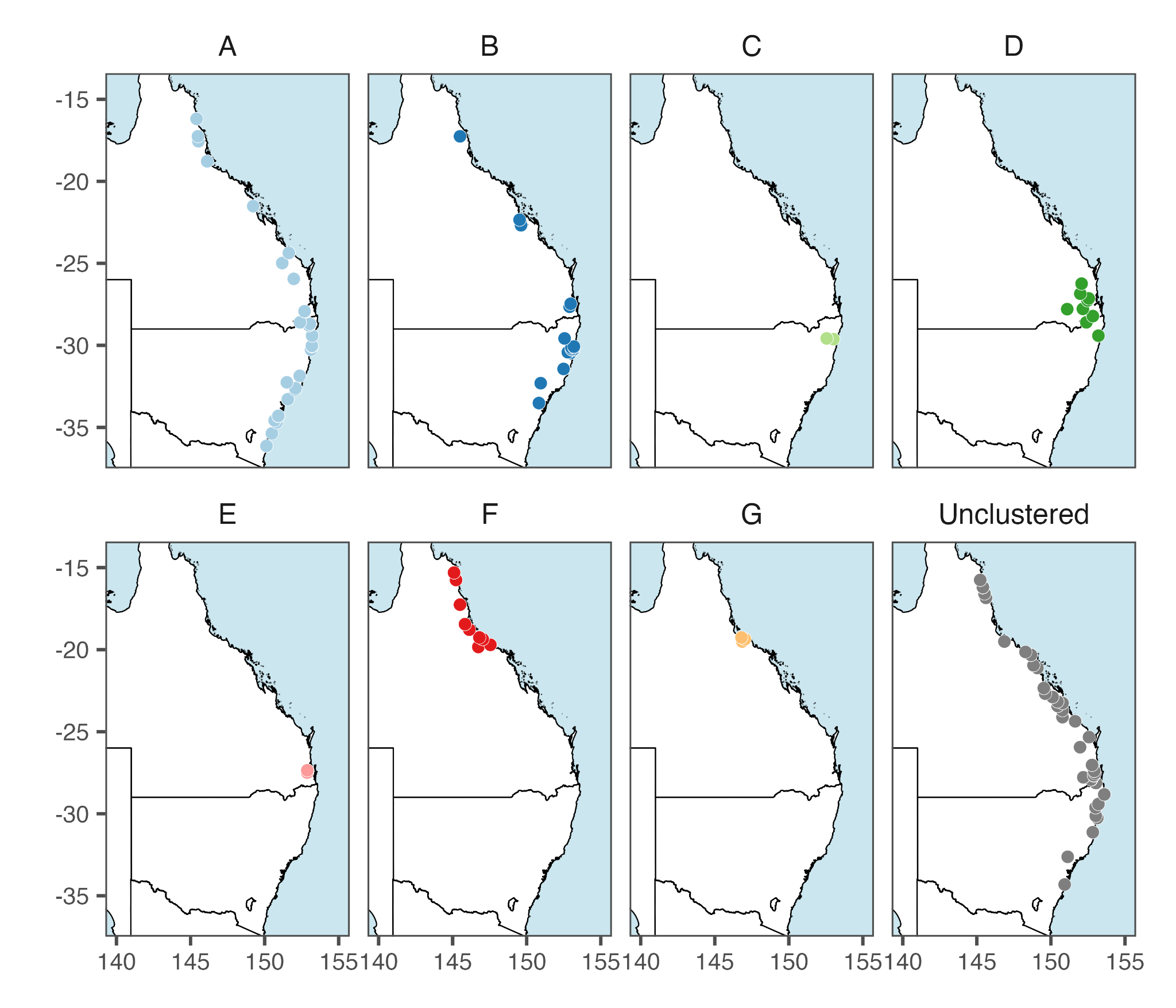
Geographic distribution of lantana samples assigned to genetic lineages based on population analysis. These occurrence points were used to construct the HSMs.

#### Fig. S3. Individual Heterozygosity


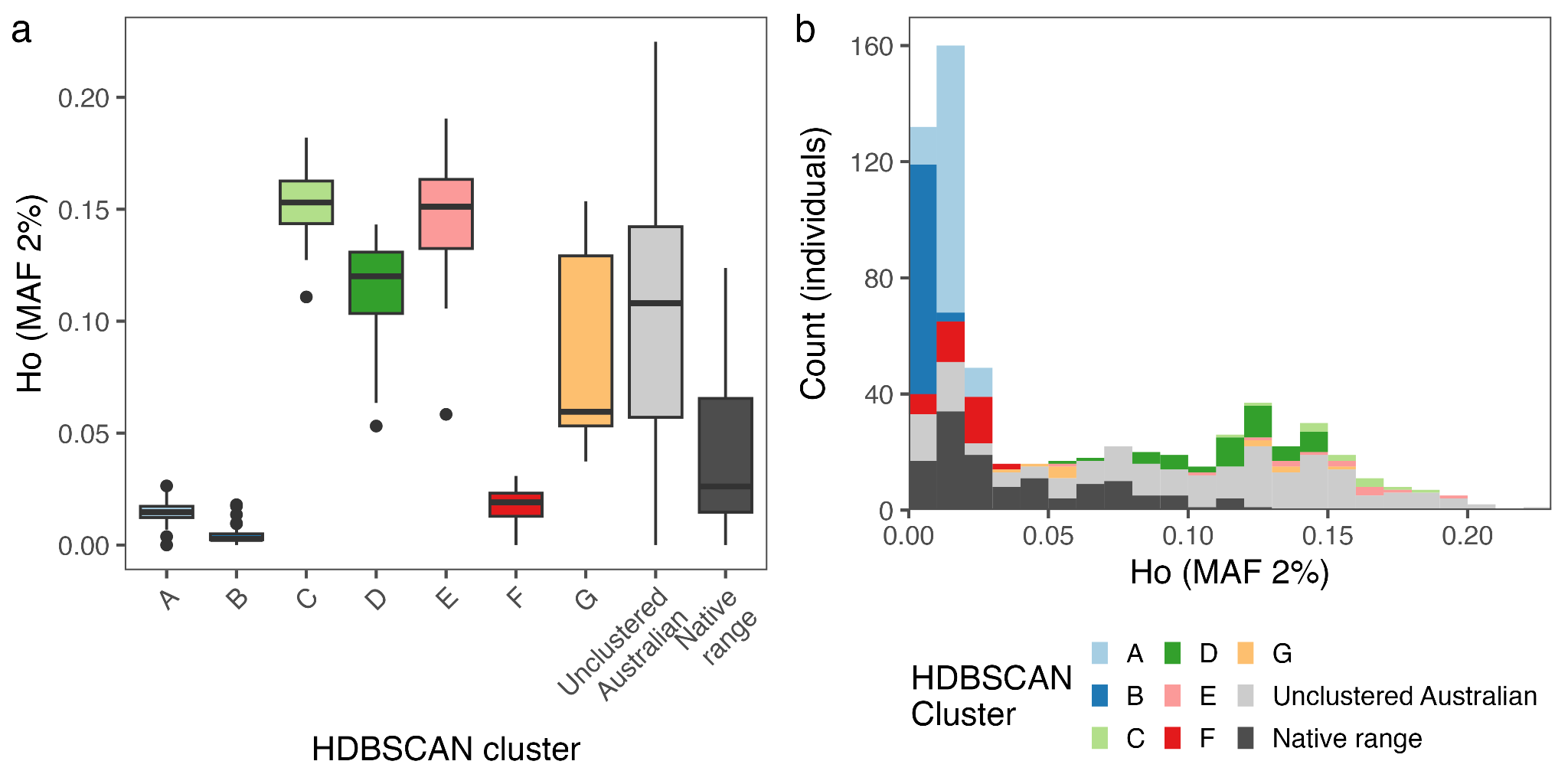
Observed heterozygosity at loci with a minimum minor allele frequency (MAF) > 2%. (A) Boxplot displaying heterozygosity by genetic lineage. (B) Histogram showing the distribution of the proportion of heterozygous loci across all samples.

#### Fig. S4. Extended LEA snmf results


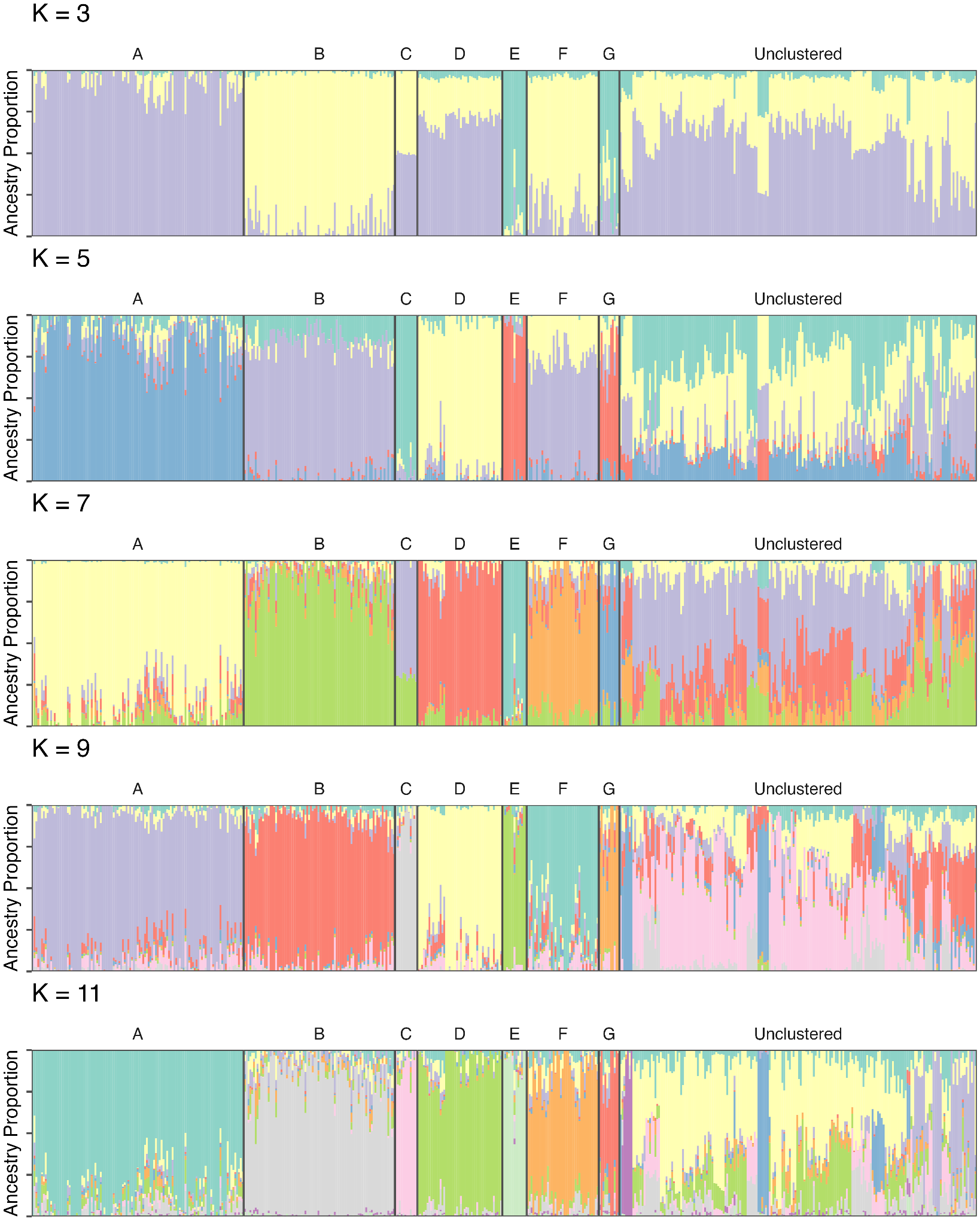
Ancestry estimation by LEA snmf for K = 3, 5, 7, 9, and 11 on eastern Australian lantana (grouped by genetic lineages); cross entropy plotted for different values of K.


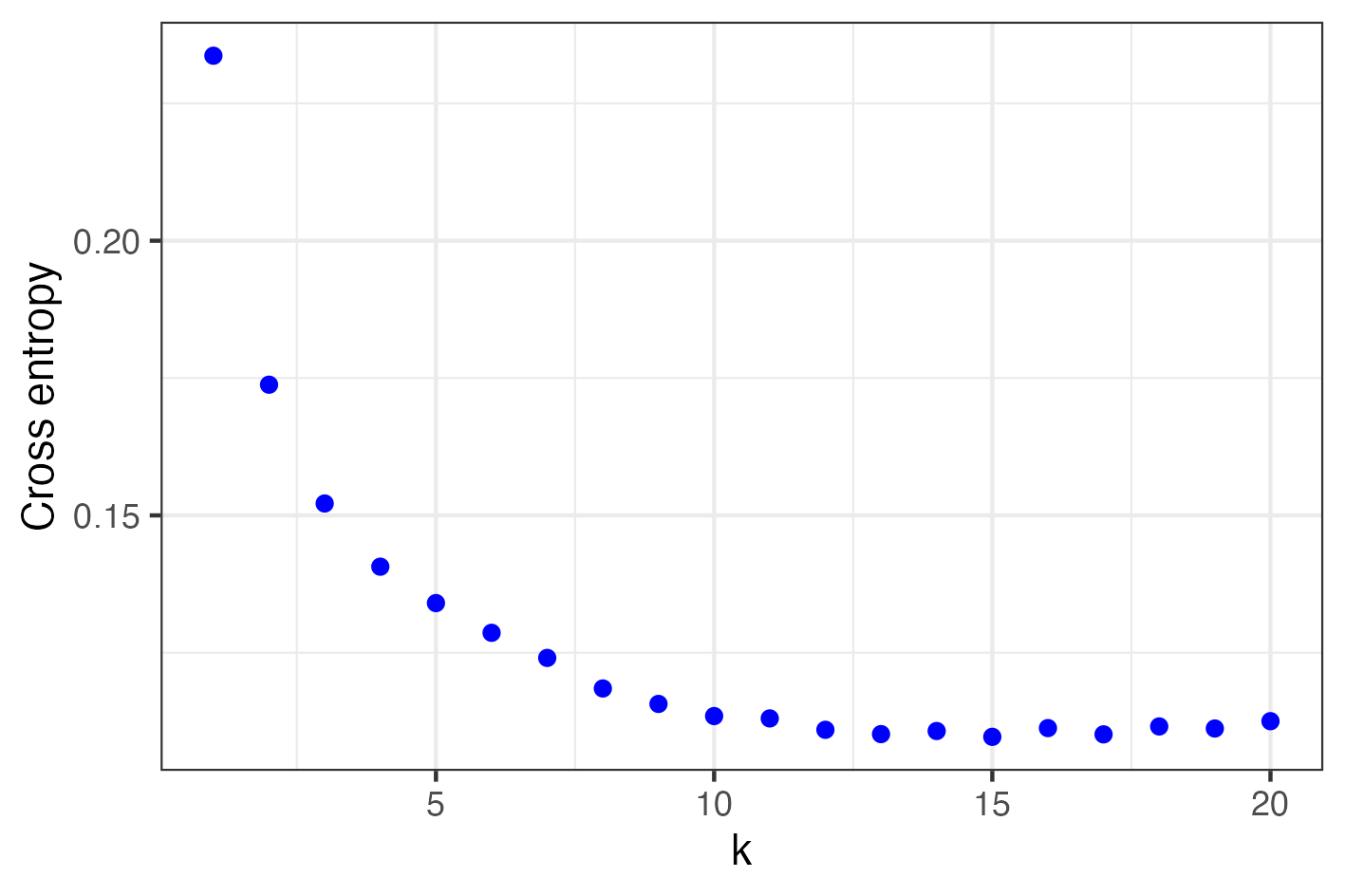


#### Fig. S5. Putative hybrid results

Hybridization evidence between Common Pink and Common Pink-Edged Red lantana (lineages A and B), based on 33 individuals from four sites (two with uniform flower colour and two with mixed/intermediate colours).

(a) Plot of the first principal component axis (PC1) vs. observed heterozygosity (4,986 loci), with putative hybrids clustering in the center. PC1 is the same as in Fig. 2a.

(b) Heatmap of loci fixed in Common Pink and Common Pink-Edged Red, with yellow = fixed reference alleles, blue = fixed alternate alleles, green = heterozygous loci, and white = missing data. Putative hybrids are predominantly heterozygous (green) at sites which are fixed for non-hybrid individuals.


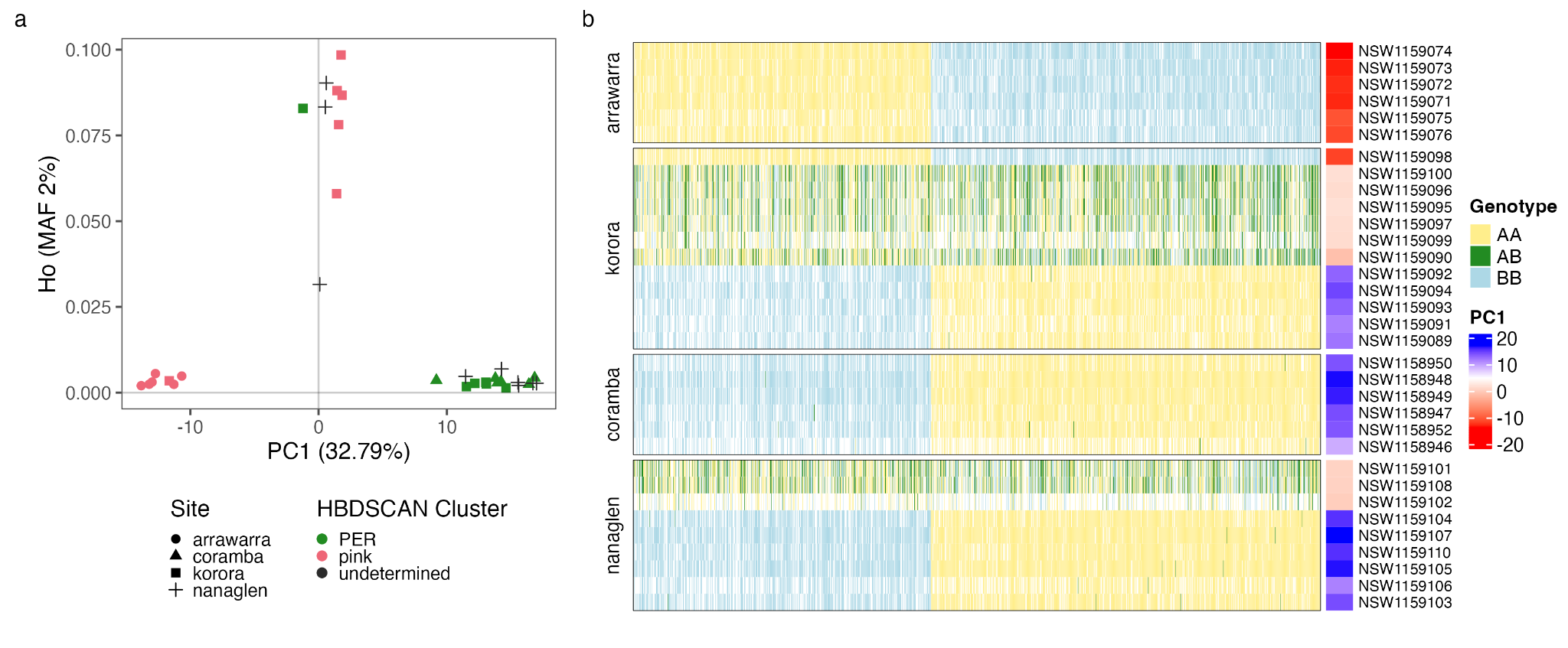
For the allele heatmap, data was filtered to retain relevant samples, remove loci with <98% reproducibility, exclude fixed loci, and keep one SNP per DArT tag. Alternatively fixed SNPs between parent groups were identified with a 5% allele frequency leniency (fixed >95%), and loci with >80% missing data were excluded, resulting in 1,455 loci.

#### Fig. S6. Habitat suitability predictions for (a) - (g) each of seven lantana genetic lineages identified; (h) all individuals sampled from invasive populations as part of this study

We note that the number of geographic occurrence records used to predict habitat suitability of genetic lineages was low due to the small number of individuals identified as belonging to each lineage, particularly in lineages C - G; we therefore suggest that these results are highly preliminary and should be used as a basis for further work.


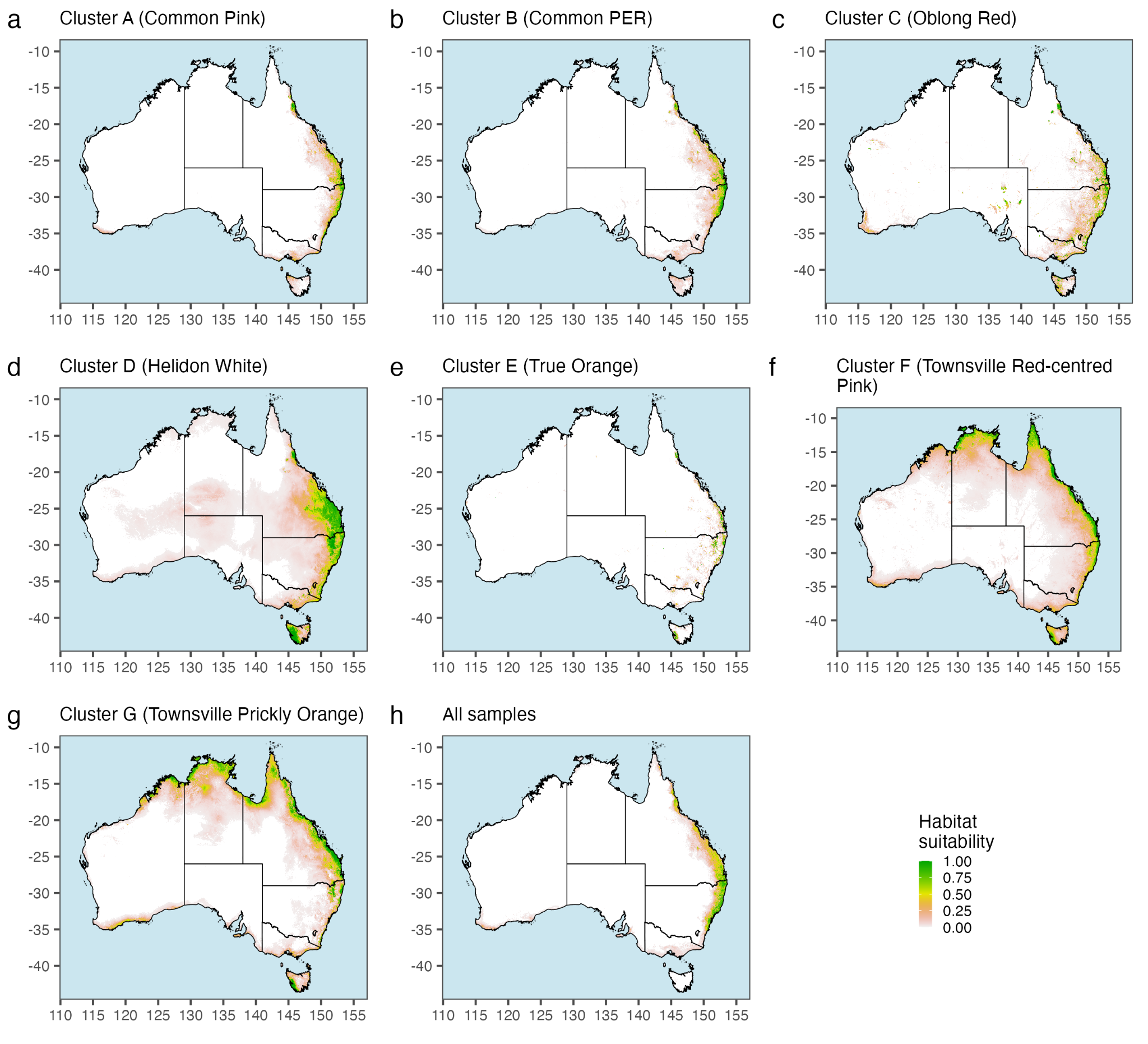


#### Additional Supplementary Materials

Response curves to environmental predictors for modelling suitable habitat for individual lantana genetic lineages, and all Australian samples combined.

[attached separately]
